# Pickering Emulsions Stabilized by Polystyrene Particles Possessing Different Surface Groups

**DOI:** 10.1021/acs.langmuir.1c02648

**Published:** 2022-01-14

**Authors:** Raojun Zheng, Bernard P. Binks

**Affiliations:** Department of Chemistry, University of Hull, Hull HU6 7RX, U.K.

## Abstract

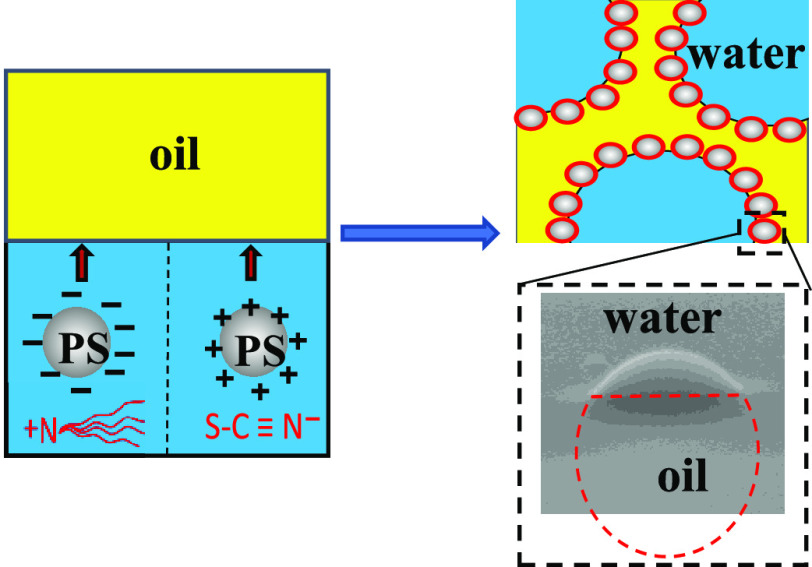

Colloidal polystyrene (PS) latex particles in water can undergo interesting charge reversal in the presence of particular electrolytes. It is worth exploring the effect of charge reversal on the properties of Pickering emulsions they stabilize. Herein, emulsions stabilized by PS latex particles possessing different surface groups (sulfate, amidine, or carboxyl) were prepared in the presence of tetrapentylammonium bromide (TPeAB) or sodium thiocyanate (NaSCN) electrolytes. The effect of salt concentration on the charge of the particles and their colloid stability was measured. Emulsions were prepared from aqueous dispersions, and their type and stability were determined. The three-phase contact angle of particles at the planar oil–water interface was also measured using a gel trapping technique. It was found that the type of emulsion stabilized by latex particles is dominated by the hydrophobic PS portion on particle surfaces, although their surface charge is strongly affected by electrolyte addition. Preferred emulsions were always water-in-oil with dodecane, and charge reversal had little influence on the emulsion type and stability. However, transitional phase inversion of emulsions stabilized by carboxyl latex particles occurred on adding salt when the oil was a low-viscosity polydimethylsiloxane.

## Introduction

Surfactant-free monodisperse polystyrene (PS) latex particle dispersions in water have long been considered as a suitable model system to study the fundamental properties of colloidal dispersions. These particles are spherical and monodisperse and can be prepared in good quantity and in a reproducible way. They are stabilized by the electrical repulsion introduced by charged groups on their surface that are covalently linked to the polymer molecules.^1^ According to the theory of Derjaguin, Landau, Verwey, and Overbeek (DLVO),^2,3^ the presence of electrolyte in the aqueous phase will screen the surface charge and lead to aggregation of the particles. However, interesting charge reversal of polystyrene particles induced by specific electrolytes has been reported.^4−12^ Anionic sulfate latex particles show positive electrophoretic mobility in the presence of tetraamylammonium cations^4^ or cationic poly(vinylamine).^5^ The symmetrical monovalent cation tetraphenyl arsonium^7^ (Ph_4_As^+^) or monovalent anion tetraphenylborate^8^ (Ph_4_B^–^) induces charge inversion of sulfonated PS particles and amidine PS particles, respectively. In addition, the charge of amidine latex particles changes from positive to negative upon addition of multivalent polyelectrolytes.^9,12^ These kinds of electrolytes all include organic counterions to that of the particles. Non-DLVO interactions, *e.g*., hydrophobic attraction, between colloidal particles have been involved in explaining the mechanism of charge reversal.^7−14^ When organic counterions accumulate near the particle surface, the hydrophobic groups adsorb onto the hydrophobic areas of particle surfaces due to the hydrophobic effect.

In addition to organic electrolytes, some inorganic salts were also reported to induce charge reversal of PS particles. Schneider *et al.*(14) found that at a high concentration, both Mg^2+^ and La^3+^ counterions accumulate at the proximity of particle surfaces leading to charge reversal. Elimelech and O’Melia^15^ found charge reversal of sulfate PS latex particles in the trivalent salt LaCl_3_. The mechanism of charge reversal induced by inorganic ions was suggested to lie in ion-specific effects,^16−19^ meaning that ions follow the same sequence regarding the capability of aggregating colloidal particles as the Hofmeister series:
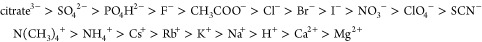
where anions (cations) to the left of Cl^–^ (Na^+^) are referred to as kosmotropic (chaotropic) ions and anions (cations) to the right of Cl^–^ (Na^+^) as chaotropic (kosmotropic) ions.^16^ Kosmotropic ions interact with water more strongly than water itself, *i.e*., they are strongly hydrated and known as structure makers. Chaotropic ions are weakly hydrated and known as structure breakers.^17^ If a hydrophobic surface, *e.g.*, polystyrene, is present, then chaotropic ions can accumulate or specifically adsorb onto this surface and result in a change of the surface potential.

Given the occurrence of charge reversal of certain PS particles in water, we thought that it is interesting to investigate their behavior at oil–water interfaces. Charged particles in water preferentially stabilize oil-in-water (o/w) Pickering emulsions and if the charge can be neutralized by some means, emulsion phase inversion to water-in-oil (w/o) ensues since uncharged particles are more hydrophobic. Thus, in systems with inorganic particles such as silica, the variation in surface charge has a dramatic influence on their properties. It is interesting to ask whether changes in the surface charge of partially hydrophobic polystyrene latex particles induce double phase inversion of emulsions they stabilize. That is, are o/w emulsions formed at low electrolyte concentrations where particles are highly charged, w/o emulsions formed at moderate electrolyte concentrations where the surface charge is neutralized, and o/w emulsions re-formed at high electrolyte concentrations where particles are oppositely charged after charge reversal? Addition of cationic surfactant induces charge reversal of silica particles and phase inversion of their particle-stabilized emulsions.^20,21^ While charge-stabilized PS latex particles have been extensively investigated at planar fluid–fluid interfaces,^22−24^ it is surprising that little exists on using these particles as an emulsifier of oil and water.^25−32^ The aim of this paper is to investigate emulsions stabilized by PS particles possessing different types of surface groups in the presence of electrolytes able to induce charge reversal. The influence of electrolyte concentration on the properties of aqueous particle dispersions is studied first.

## Experimental Section

### Materials

Water was purified using an Elga Prima reverse osmosis unit and then treated with a Milli-Q reagent system and had a resistivity of 18 MΩ cm. Dodecane (≥99%), sodium thiocyanate (NaSCN, ≥99.99%), tetrapentylammonium bromide (TPeAB, ≥99%), and 1 cS polydimethylsiloxane (PDMS) were purchased from Aldrich. The latter oil is an oligomer. Potassium chloride (KCl, >99%), sodium hydroxide (NaOH, >98.6%), and hydrochloric acid (HCl, 37.4%, analytical grade) were purchased from Fisher Scientific. Prior to use, dodecane and PDMS were passed through a column of chromatographic alumina (Merck kGaA, particle size: 0.063–0.200 mm) to remove polar impurities. Surfactant-free PS latex particles possessing different surface groups (sulfate, amidine, or carboxyl) were purchased from Invitrogen Corp. (Thermo Fisher Scientific) and received as aqueous dispersions. Anionic sulfate (SO_4_^2–^) particles were received at a particle concentration of 8 w/v%, and cationic amidine (C(*NH*)NH_3_^+^) and anionic carboxyl (COO^–^) particles were received at 4 w/v%. The particles are stabilized against aggregation by covalently linked charged groups. The charged ends take up less than 10% of the particle surface area. Hence, the surface of these particles is relatively hydrophobic. According to the manufacturer, these particles were cleaned by dialysis against deionized water until the conductance is close to that of deionized water. Nano-sized particles (typically 0.2 μm) were used to stabilize emulsions, whereas micron-sized particles were used to measure the contact angle of particles at the planar oil–water interface using a gel trapping technique (GTT), with the assumption that carefully selected small and large particles have similar surface properties. To achieve this, we deliberately purchased micron-sized particles based on their surface charge density to match it as close to the corresponding nano-sized particles as possible. Figure S1 presents SEM images of the three types of micron-sized latex particles. Particles were characterized by the manufacturer, and some properties are listed in Table 1. Gellan gum was kindly provided by CPKelco (USA). It is a food-grade gel consisting of tetrasaccharide repeat units (d-glucose, d-glucuronic acid, d-glucose, and l-rhamnose).^33^ It can be hydrated in water and forms single coils at high temperatures (>90 °C). By lowering the temperature, the disordered coils aggregate into ordered double helices and finally cross-link into a gel. Ethylenediaminetetraacetic acid disodium salt (EDTA disodium salt, >99%, Sigma-Aldrich) and acetonitrile (Honeywell, ≥99.9%) were used as received. PDMS Sylgard 184 elastomer and its curing agent produced by Dow Corning (USA) were purchased from Ellsworth Adhesives Ltd. (UK).

**Table 1:** Properties of PS Latex Particles Given by the Manufacturer

particle surface group	particle diameter/μm	no. surface groups/particle	area per surface group/Å^2^	surface charge density/mC m^–2^
sulfate	0.25 ± 0.008	6.8 × 10^3^	2906	–6
	1.9 ± 0.052	1.8 × 10^6^	637	–25
amidine	0.22 ± 0.010	1.2 × 10^5^	122	+132
	1.0 ± 0.044	3.1 × 10^6^	103	+156
carboxyl	0.21 ± 0.007	1.1 × 10^5^	123	–103
	3.6 ± 0.25	3.3 × 10^7^	124	–129

### Methods

#### Preparation and Characterization of Aqueous Particle Dispersions

Batches of PS latex particle dispersions were prepared through dilution of the stock dispersion using Milli-Q water or the corresponding salt solution. Their pH was adjusted using acid (0.1 M HCl) or base (0.1 M NaOH) and measured using a Jenway-3510 pH Meter with an InLab Flex-Micro electrode (Mettler-Toledo Ltd). The zeta potential of particle dispersions was measured at 25 °C with a Zetasizer Nanoseries Nano ZS (Malvern Instruments) equipped with a 4 mW He–Ne laser beam operating at λ = 633 nm. Measurements were made by introducing a universal dip cell (ZEN1002, Malvern Instruments) inside a plastic disposal cuvette (1 cm path length). Three measurements of 12 runs were conducted for each sample. The particle concentration for this measurement is 0.0008 wt % for sulfate latex particles and 0.0004 wt.% for amidine and carboxyl latex particles. Particle dispersions are transparent at such low concentrations even in the presence of electrolyte. The vessels containing particles were shaken gently before sampling. The volume weighted diameter distribution of particles at different salt concentrations was determined by light diffraction using a Malvern Mastersizer 2000 instrument fitted with a small volume sample dispersion unit. The instrument was thoroughly cleaned by adding anhydrous ethanol to the sample dispersion unit and stirred at 2000 rpm three times followed by addition of Milli-Q water at the same stirring speed three times. The background was then measured after which ∼0.5 mL of particle dispersion was added to the sample dispersion unit containing 150 mL of Milli-Q water. The refractive index of the particles and water is 1.590 (polystyrene at λ = 500 nm, 20 °C) and 1.333, respectively. The size range is 0.02–2000 μm, and the analysis model is the Spherical General Purpose one.

#### Preparation and Characterizations of Emulsions

Equal volumes (2.5 mL) of PS latex particle dispersion and dodecane were added to a screw-top glass vial of inner diameter 1.6 cm and height 7.2 cm. The two phases were emulsified using an IKA Ultra Turrax T25 homogenizer with an 8 mm head operating at 13,000 rpm for 2 min. All the experiments were conducted at room temperature (22 ± 1 °C). The emulsion type was inferred from the drop test and conductivity measurement. If the conductivity of an emulsion is close to that of the aqueous phase, then the emulsion is o/w. If the conductivity is similar to that of oil, then the emulsion is w/o. Photos of the obtained emulsions were taken immediately after preparation and with time to visually evaluate their stability. Optical microscopy of the prepared emulsions was taken using an Olympus BX-51 microscope fitted with a DP70 digital camera and Image-Pro Plus 5.1 software (Media Cybernetics) by placing a drop of emulsion on a glass microscope slide. The mean droplet diameter was calculated with Image J 1.47v. If the number of droplets is less than 50, then all the droplets were used, otherwise between 50 and 100 droplets were used.

#### Measurement of Contact Angle Using the Gel Trapping Technique

The GTT first reported by Paunov^34^ was used to measure the three-phase contact angle of single particles at a planar oil–water interface with some modification. The technique is based on spreading particles at an oil–water interface and subsequently gelling the aqueous phase with gellan gum. With the particle monolayer trapped on the surface of the aqueous gel, the oil was removed and replaced by PDMS elastomer, which allows the exposed surface of the particles to embed within the polymer. After curing, the PDMS layer was peeled carefully from the gelled aqueous phase such that particles embedded within it can be imaged with high-resolution scanning electron microscopy (SEM). The position of the particles relative to the PDMS surface and the diameter of the contact line of the particles with the PDMS surface can be determined from the SEM images, enabling the particle contact angle to be determined. The GTT method is applicable to particles with diameters in the range of several hundred nanometers to a few hundred micrometers^34^ and has also been used in the presence of salts.^25^

Prior to use, gellan gum was purified according to the method reported by Paunov^34^ and dried by evaporation. Batches of electrolyte solutions at various concentrations were prepared at the required pH, and a known amount of solid purified gellan was added to the solutions and heated to 95 °C in a water bath for 30 min to obtain 3 wt % gellan-salt solutions. The gellan gum concentration used by Paunov^34^ was 2 wt %, which we found to be too soft after gelation. A large fraction of gellan gum peeled off together with latex particles after curing. Hence, we increased the gellan gum concentration to 3 wt %. The gellan gum-salt solutions were then kept in an oven at 70 °C for further use. Aqueous suspensions of 1 wt % latex particles were prepared by mixing stock dispersions with 2-propanol (water:2-propanol = 50:50 by vol), which was used as a spreading solvent. Oil was prewarmed to 70 °C to match the temperature of the purified gellan solution. Then, 2.5 mL of hot gellan-salt solution was poured into a preheated plastic Petri dish of diameter 35 mm and the same amount of pre-heated oil was carefully introduced on top of the gellan solution. A small sample (typically 10 μL) of latex particle suspension in the spreading solvent was carefully spread at the oil–water interface using a 100 μL syringe. The Petri dish including the sample was kept at room temperature for 1 h to allow gelling of the aqueous phase. Afterward, the oil phase was decanted and any residue was carefully removed from the edge of the Petri dish using tissue paper. PDMS Sylgard 184 was mixed with its curing agent in the ratio 10:1 (by mass) and centrifuged at 3000 rpm for 5 min to remove any air bubbles formed during mixing. Subsequently, 2.5 g of PDMS Sylgard 184-curing agent was carefully layered over the gelled aqueous phase with the particle monolayer to avoid trapping of air bubbles and was cured at least for 48 h at room temperature. Figure S2 shows the process of removing the PDMS Sylgard 184 containing attached particles from the gelled aqueous layer, after which it was incubated in hot aqueous solutions of 20 mM EDTA disodium salt, 20 mM sodium hydroxide, and Milli-Q water for 5 min to wash off any gellan residues.

A Carl Zeiss EVO-60 SEM instrument with a secondary electron detector was used to image particle monolayers on PDMS Sylgard 184 at a voltage of 20 kV and a probe current of 100 pA. Before imaging, samples were coated with an ∼10 nm carbon layer (spectrally pure graphite) using an Edwards high vacuum evaporator. The three-phase contact angle θ of the particles measured into water was determined from the SEM micrographs using the following analysis:

(i) If the particle contact line diameter *d*_c_ is below the particle equatorial diameter *D* (hydrophilic particles, θ < 90°), θ was determined from
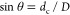
1
(ii) For hydrophobic particles, θ > 90°, whose contact line is above the particle equatorial diameter, θ was calculated from
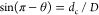
2

## Results and Discussion

### Systems with Sulfate Latex Particles

**Figure 1:**
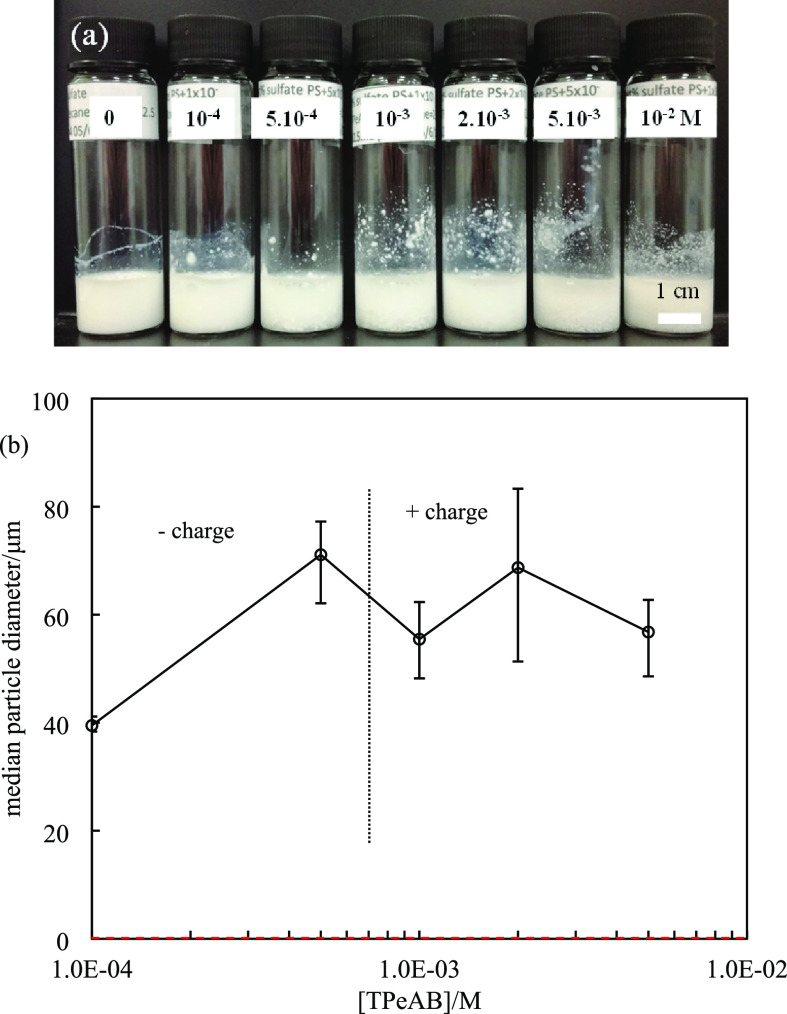
(a) Appearance of 2 wt % sulfate latex particle dispersions (diameter = 0.2 μm) in the presence of TPeAB at pH = 4 taken immediately after preparation. (b) Average diameter and charge type of particles in (a); the red dashed line shows the average diameter of particles without salt (0.15 μm).

**Figure 2:**
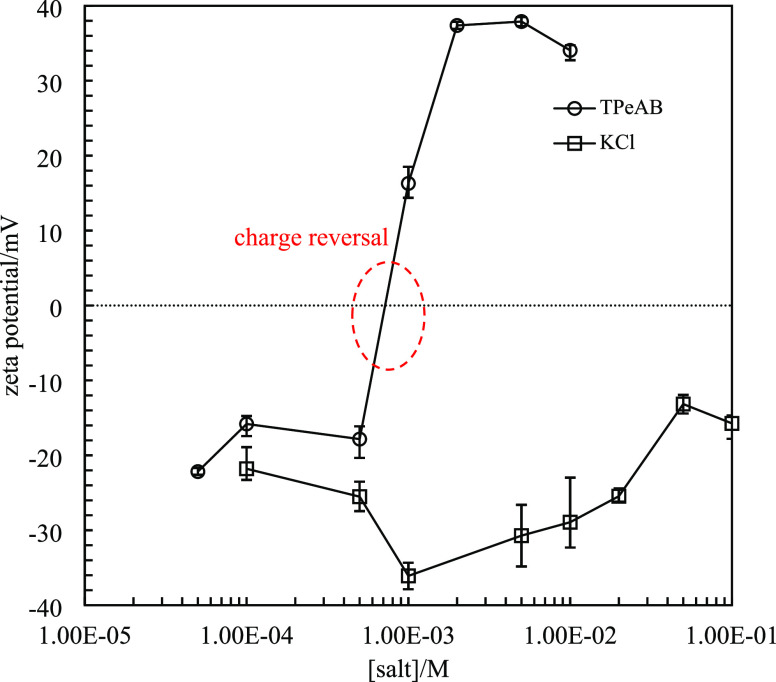
Zeta potential of 0.0008 wt % sulfate latex particles (diameter = 0.2 μm) in the presence of TPeAB or KCl at different concentrations at pH = 4. The zeta potential in pure water is −40 mV.

Prior to investigating the properties of emulsions stabilized by latex particles, the properties of aqueous particle dispersions were studied. The pH was maintained at 4 to avoid effects due to the dissolution of carbon dioxide.^35,36^ Particles are negatively charged regardless of pH due to the presence of fully ionized sulfate groups. Figure 1a shows the appearance of 2 wt % particle dispersions at varied TPeAB concentrations. Particles are initially discrete in pure water. When 1 × 10^–4^ M TPeAB is added, particles flocculate immediately and large aggregates are visually observed. Figure 1b represents the median particle diameter as a function of salt concentration. The average diameter of sulfate PS latex particles in pure water is 0.15 μm. A log normal distribution corresponding to a single particle population centered between 0.1 and 0.2 μm is observed (Figure S3). Addition of 1 × 10^–4^ M TPeAB results in an aggregate diameter of 40 μm, with a wide distribution ranging from 1 to 200 μm. Figure 2 shows the zeta potential of 0.0008 wt % sulfate PS latex particles in TPeAB and, for comparison with an inert electrolyte, KCl solutions. As can be seen, the absolute values increase with the increasing KCl concentration, reach a maximum, and then decrease. Similar maxima have been reported before, and different models have been proposed to explain this phenomenon.^15,37,38^ The hairy layer model^38^ assumes that the latex particle is covered by a layer of polymer chains (“hairs”) with terminal end groups. Without electrolyte, the polymer chains expand due to repulsion between charged groups. When electrolyte is added, the hairs contract due to screening of charges, resulting in the position of the shear plane moving inward; the zeta potential is therefore higher. Further increase in electrolyte concentration screens the charge on particle surfaces resulting in a decrease in the zeta potential. The co-ion adsorption model^15^ suggests that, at low electrolyte concentrations, co-ions like Cl^–^ adsorb to particle surfaces, increasing the surface charge to a maximum. At high electrolyte concentrations, however, the particle surface is saturated with adsorbed ions and further increase in the electrolyte concentration increases the ionic strength in the dispersion. As a result, counterions screen the surface charges and lead to a decrease in the zeta potential.

In contrast to the case with KCl, a trace amount of TPeAB (5 × 10^–5^ M) significantly decreases the zeta potential from −40 to −22 mV. Further increase in salt concentration results in a continuous decrease in the absolute value until charge reversal occurs between 5 × 10^–4^ M and 1 × 10^–3^ M, after which particles become increasingly positively charged. According to the DLVO theory, addition of electrolyte screens the surface charge of particles, suppressing the double-layer repulsion and causes aggregation in colloids.^2,3^ Particles tend to aggregate faster near the isoelectric point (IEP) and become stable away from it. However, in our system, large flocs of particles are observed even at the highest salt concentration. If TPeA^+^ ions adsorb onto particles merely by electrostatic attraction, then particles should be discrete above the IEP as they are now highly charged. The formation of large flocs above the IEP must be related to another attractive interaction. It is suggested that the hydrophobic effect, which is beyond the DLVO framework, should be involved in the interaction between PS particles and organic electrolytes. Hydrophobic groups on the counterions strongly interact with the particles, resulting in their aggregation at high salt concentrations. A similar scenario has been observed in systems with tetraphenylarsonium ions and sulfonated latex particles.^4,7^ We also see that, unlike KCl, the zeta potential of particles in TPeAB does not display a maximum. This is probably also due to the strong hydrophobic adsorption of TPeA^+^ cations on negatively charged particle surfaces. The corresponding strongly diminished repulsion between the surface groups causes the hairy layer to shrink. Since the influence of shear plane movement caused by charge screening is minor, the maximum in the zeta potential is eliminated as a result.^4^

**Figure 3:**
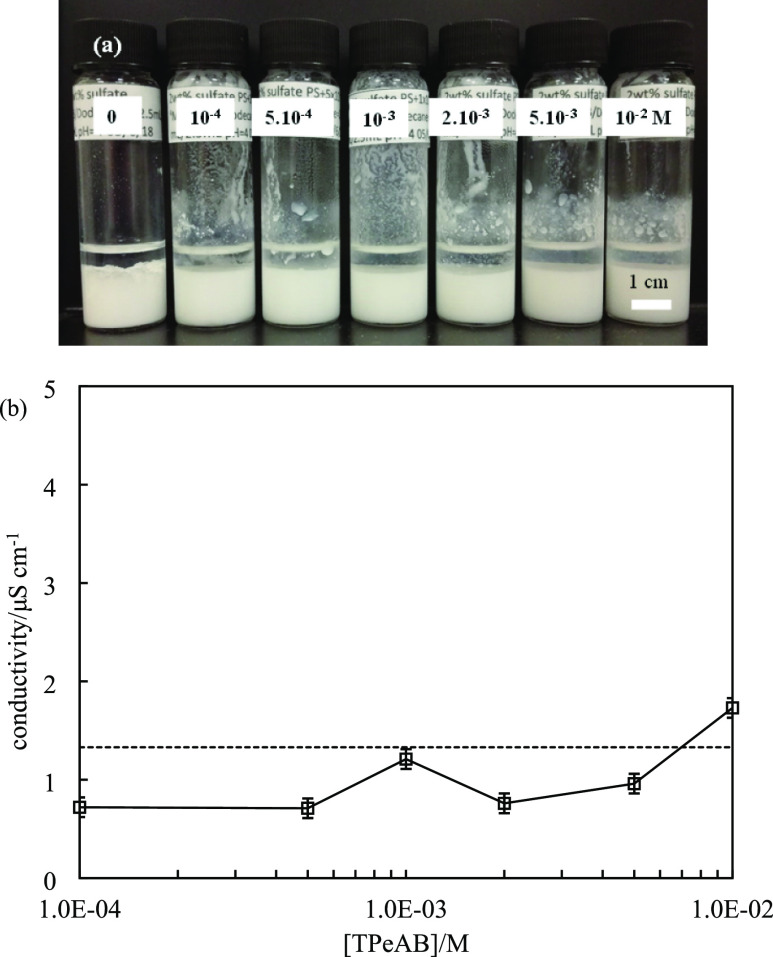
(a) Appearance of water-in-dodecane emulsions (ϕ_*w*_ = 0.5) stabilized by 2 wt % sulfate latex particles of diameter = 0.2 μm at different concentrations (given in M) of TPeAB at pH = 4 taken 1 week after preparation. (b) Conductivity of emulsions in (a) measured immediately after homogenization; the dashed line is the conductivity of emulsion without salt.

**Figure 4:**
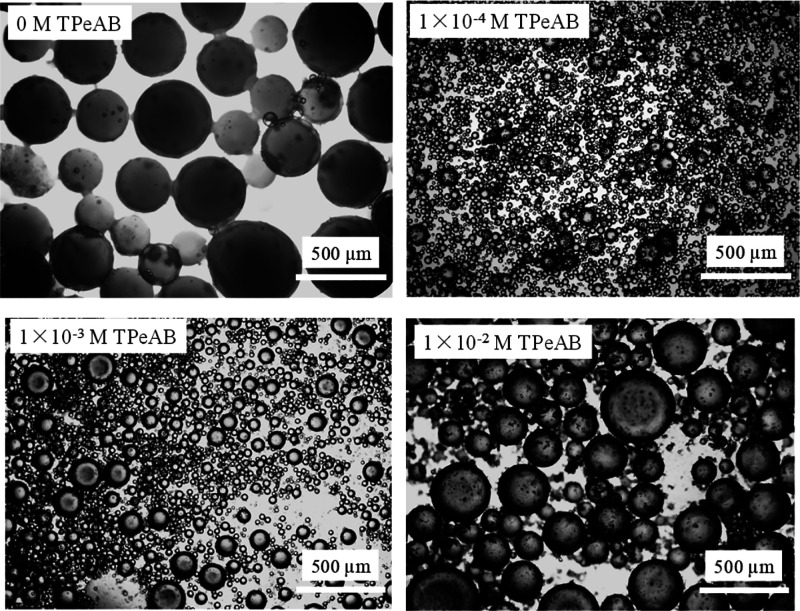
Optical microscopy images of water-in-dodecane emulsions (ϕ_*w*_ = 0.5) stabilized by 2 wt % sulfate latex particles (diameter = 0.2 μm) in the presence of TPeAB at pH = 4 taken 2 days after preparation.

**Figure 5:**
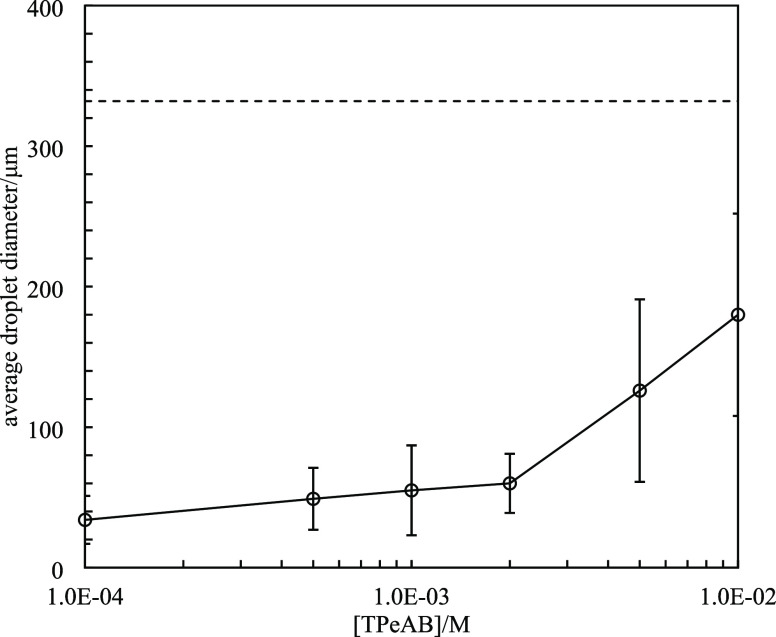
Average droplet diameters of water-in-dodecane emulsions stabilized by 2 wt % sulfate latex particles (diameter = 0.2 μm) *versus* [TPeAB] at pH = 4. The dashed line represents the average diameter of emulsion without TPeAB.

Batches of emulsions were prepared by homogenizing 2.5 mL of 2 wt % sulfate latex particle dispersion in the presence of TPeAB at pH = 4 with 2.5 mL of dodecane. The appearance of emulsions is shown in Figure 3a. Their conductivities are very low as shown in Figure 3b, indicating that the emulsions are all w/o (supported by drop tests). They sediment quickly but remain stable to coalesce for more than 6 months. Optical microscopy images of selected emulsions are shown in Figure 4, in which spherical droplets exist throughout. Nothing dramatic occurs on passing through the salt concentration where particles in water exhibit charge reversal. Figure 5 is a plot of the average water droplet diameter as a function of salt concentration. Without salt, droplets of average diameter 330 μm are obtained, indicating that even though sulfate latex particles can stabilize emulsions, the droplets are quite large. When TPeAB is added, a dramatic decrease in the droplet size is observed. Adding 1 × 10^–4^ M salt results in droplets of 35 μm in diameter. The droplet size then increases progressively with salt concentration. The increase at high salt concentrations is probably due to the charge reversal of particles, where particles are increasingly positively charged. We recall that an average particle floc size higher than 40 μm was determined on adding TPeAB to aqueous suspensions. In some of the produced emulsions, an average droplet size of ∼50 μm was observed. If particles adsorb on the droplet surface in the form of flocs, then much larger droplets should be expected.^26^ One possibility is that the presence of TPeAB decreases the oil–water interfacial tension, assisting the stabilization of smaller water droplets. This is reasonable because TPeAB has been shown to decrease the air–water surface tension to ∼50 mN/m at around 1 mM.^39,40^ Another possibility is that the high-speed shearing during homogenization breaks particle flocs into smaller aggregates (or even single particles) temporarily.^27^

**Figure 6:**
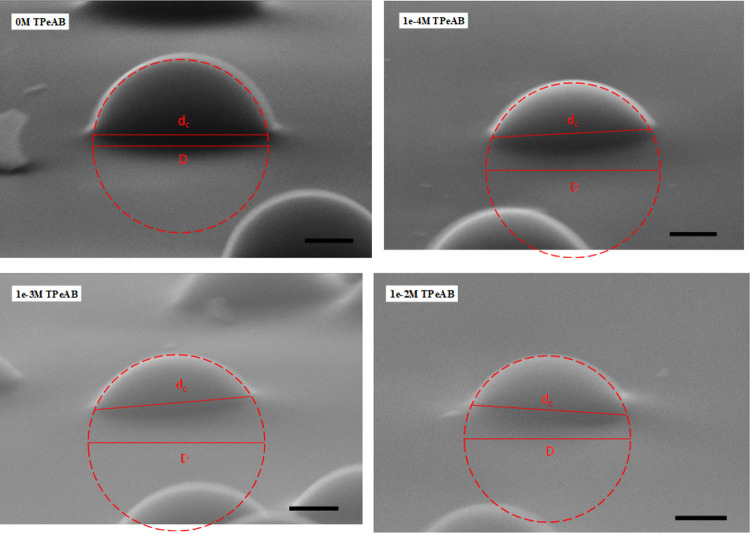
SEM images of monolayers of monodisperse sulfate latex particles (diameter = 2 μm) on the surface of PDMS Sylgard 184 at the dodecane–water interface at different [TPeAB]. Observation angle δ = 80°, scale bar = 500 nm. The visible part of particle surfaces was immersed in water, while the particle surface immersed in elastomer was originally in oil. The particle contact line diameter, *d*_c_, can be measured directly from the images, and the particle equatorial diameter, *D*, is obtained by extrapolation after fitting the particle profile with a circle.

**Figure 7:**
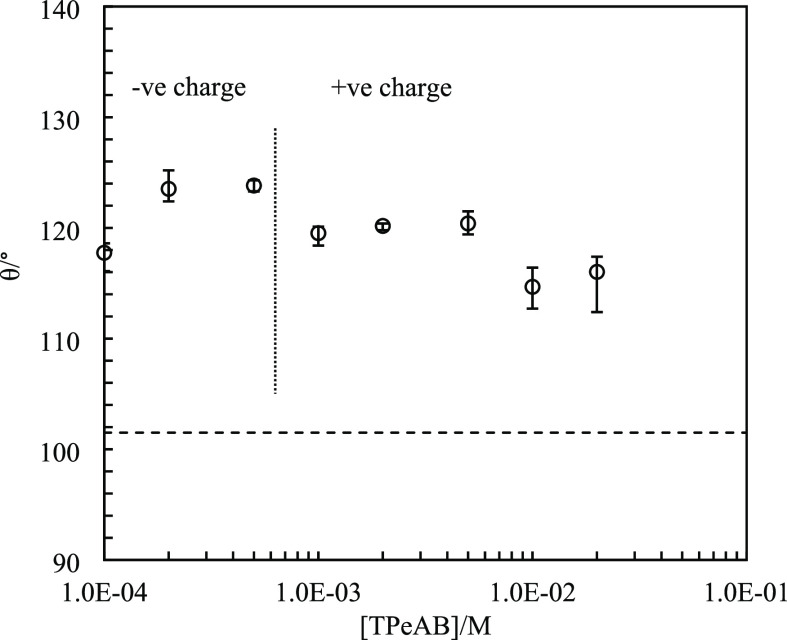
Three-phase contact angle (θ) of sulfate latex particles (diameter = 2 μm) at the dodecane–water interface *vs* [TPeAB] at pH = 4. The horizontal dashed line indicates contact angle without salt.

As all w/o emulsions were obtained in this system, it is necessary to measure the three-phase contact angle of sulfate latex particles at the planar dodecane–water interface. The GTT was used for the measurement, and particles with a diameter of 2 μm were chosen. It is assumed that large and small particles used here have a similar wettability as their surface charge densities are close: −25 mC/m^2^ for 2 μm particles and – 6 mC/m^2^ for 0.2 μm particles. Figure 6 shows SEM images of the particles gel-trapped and micro-cast with PDMS Sylgard 184 at different concentrations of TPeAB. The visible fraction of particle surfaces was immersed in the aqueous phase, while the particle surface immersed in elastomer PDMS was originally in the oil phase. The particle contact angle was calculated using eqs 1 or 2 after measuring the contact line diameter of individual particles from the images, *d*_c_, and fitting a circular profile on the particles to determine their equatorial diameter, *D*. The contact angle is plotted in Figure 7 as a function of [TPeAB]. The value at the dodecane–water interface without salt is 101 ± 5°, slightly lower than that of 120 ± 12° for sulfate latex particles (diameter: 1.6 μm) at the octane–water interface.^41^ Addition of 1 × 10^–4^ M TPeAB results in an increase in contact angle to 118°, after which it decreases slightly with the salt concentration although all of them are ≥115°. This is interesting as the addition of organic electrolyte usually results in an increase in particle hydrophobicity. Counterions adsorb on the particle surface by electrostatic attraction, exposing hydrophobic chains to water. However, in our case, as there is a combination of electrostatic attraction and hydrophobic attraction between TPeA^+^ ions and particle surfaces, the effect on particle wettability is more complicated. A schematic of the different possible arrangements of TPeA^+^ ions on the surface of sulfate latex particles is shown in Figure S4a. At low salt concentrations, TPeA^+^ ions absorb onto negatively charged sites on particle surfaces by electrostatic attraction with the hydrophobic chains exposed to water, leading to an increase in hydrophobicity. When charge neutralization is achieved, the hydrophobic interaction between TPeA^+^ ions and the bare polystyrene surface dominates such that the charged nitrogen ion becomes exposed to water, resulting in a decrease in hydrophobicity. Since θ is >90° in all cases, it is predicted that emulsions would be w/o as we find experimentally.

This system exhibits two intriguing properties. First, the particles are partially hydrophobic but they are initially dispersed in water due to the presence of charged sulfate groups. The PS portion gives particles a hydrophobic character, whereas sulfate groups contribute to the hydrophilic character. It seems that there is a competition between the PS surface and charged surface groups in dictating the preferred emulsion type. For these particles, the hydrophobic PS portion apparently dominates the emulsion type as w/o emulsions are stabilized regardless of the sign of the surface charge or the concentration of the added electrolyte. This is reasonable as sulfate groups occupy only ∼5% of the particle surface, while 95% of it is ion-free PS. Similar w/o emulsions have been reported by Golemanov *et al.*(27) with sulfate PS latex particles in the presence of NaCl. Second, unlike typical emulsions (Pickering or surfactant-stabilized) where the emulsifier is dispersed in the continuous phase, in emulsions stabilized by sulfate PS latex particles, the emulsifier is contained within the dispersed phase. Golemanov *et al.*(27) suggested that such w/o emulsions are anti-Bancroft type because the stabilizer is in the dispersed phase, while Bancroft’s rule^42^ states that “a hydrophile colloid will tend to make water the dispersing phase, while a hydrophobe colloid will tend to make water the disperse phase”. In Pickering emulsions, particles dispersed in water are not necessarily hydrophilic. If we decide whether particles are hydrophilic or hydrophobic depending on where particles are dispersed, *i.e*., particles dispersed in water are hydrophilic, while those dispersed in oil are hydrophobic, then the w/o emulsions we obtain here are anti-Bancroft type (we note however that hydrophilic particles can disperse in oil). However, if our decision is based on the three-phase contact angle, then Bancroft’s rule may be interpreted as “particles more wetted by water (hydrophilic) should stabilize o/w emulsions, and those more wetted by oil (hydrophobic) stabilize w/o emulsions”. In this case, w/o emulsions preferred here follow Bancroft’s rule.

### Systems with Carboxyl Latex Particles

**Figure 8:**
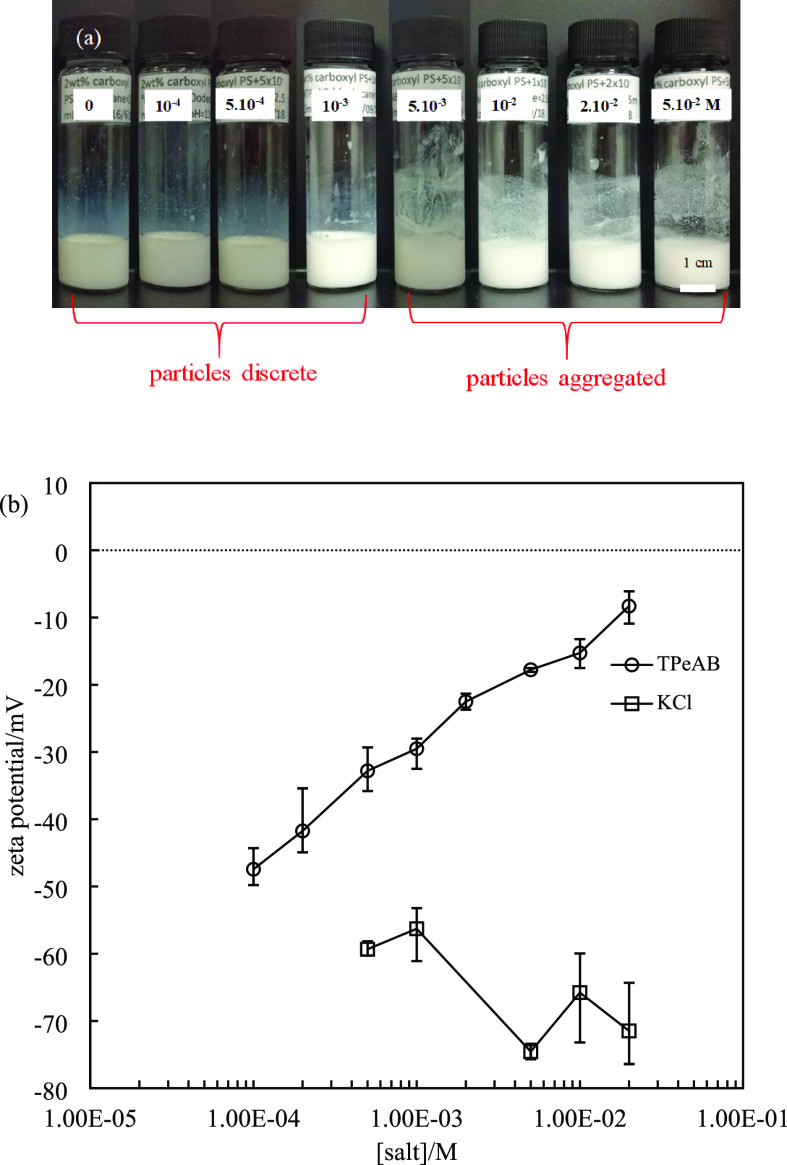
(a) Appearance of 2 wt % carboxyl latex particle dispersions (diameter = 0.2 μm) at various concentrations (given in M) of TPeAB at pH = 11 taken immediately after preparation. (b) Zeta potential of 0.0004 wt % carboxyl latex particles in KCl or TPeAB solutions at pH = 11.

The surface charge density of sulfate latex particles used in the emulsions above is relatively low (−6 mC/m^2^), and sulfate groups only occupy ∼5% of particle surfaces. We wondered whether we could observe phase inversion of emulsions using particles possessing a higher density of surface groups. Unfortunately, we were unable to purchase sulfate latex particles with higher charge densities, so we used 0.2 μm carboxyl latex particles instead. The surface charge density of the latter is −103 mC/m^2^ when fully ionized, arising from the deprotonation of the carboxyl groups. The p*K*_a_ of carboxyl groups on the surface of PS latex particles is 4.9, and the zeta potential of carboxyl latex particles remains relatively constant at pH > 9.^10,13^ Therefore, carboxyl latex particle dispersions were prepared at pH = 11 to ensure full deprotonation of carboxyl groups. The appearance of particle dispersions in the presence of TPeAB is shown in Figure 8a. Particles are discrete in water at low salt concentrations up to 5 × 10^–4^ M. Large aggregates can be observed with optical microscopy at 5 × 10^–3^ M TPeAB and above (see Figure S5), much larger than those of sulfate latex particles since the surface charge density of the latter is much smaller. The zeta potentials of 0.0004 wt % carboxyl latex particles were measured in both KCl and TPeAB solutions at pH = 11 and are shown in Figure 8b. In pure water, the zeta potential is −52 mV and its absolute value increases with the KCl concentration. Addition of TPeAB, however, results in a gradual decrease in the magnitude of the zeta potential, but no charge reversal was observed up to 2 × 10^–2^ M salt. Charge reversal of these particles may be expected at higher TPeAB concentrations, although this is experimentally challenging to determine.

**Figure 9:**
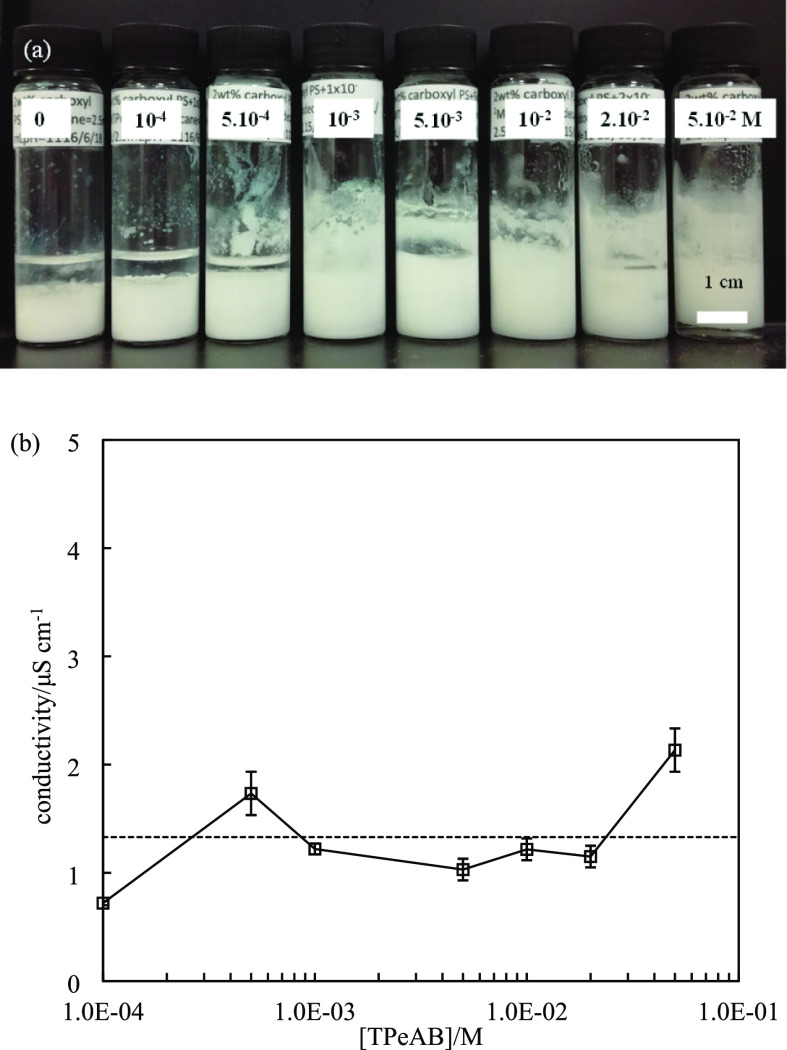
(a) Appearance of water-in-dodecane emulsions (ϕ_*w*_ = 0.5) stabilized by 2 wt % carboxyl latex particles (diameter = 0.2 μm) at various concentrations (given in M) of TPeAB at pH = 11 taken 1 week after preparation. (b) Conductivity of emulsions in (a) measured immediately after homogenization; the dashed line is the conductivity of emulsion without salt.

**Figure 10:**
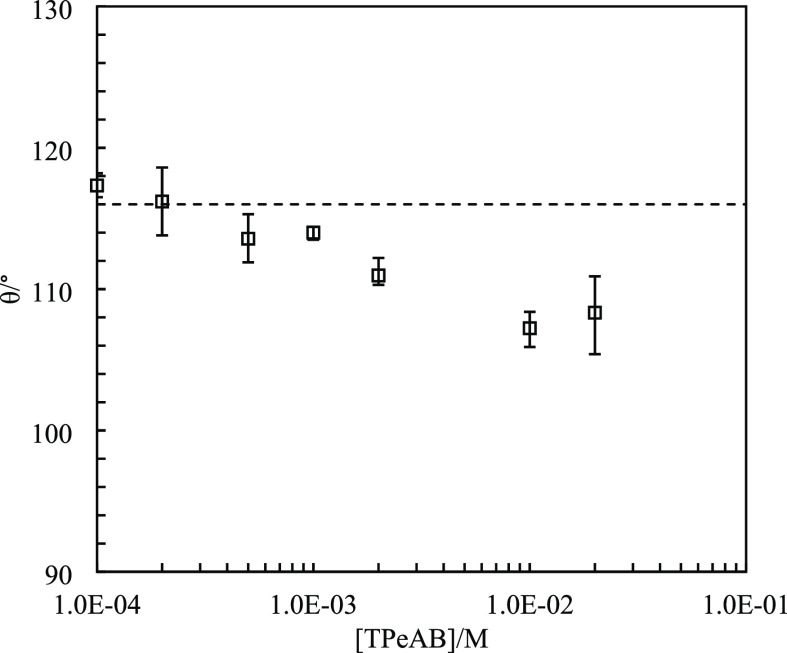
Three-phase contact angle (θ) of carboxyl latex particles (diameter = 3.5 μm) at the dodecane–water interface at pH = 11 *vs* [TPeAB]. The dashed line indicates the contact angle without salt.

Batches of emulsions were prepared by homogenizing 2.5 mL of 2 wt % carboxyl PS latex dispersion (*d* = 0.2 μm) with 2.5 mL of dodecane, and their appearance is shown in Figure 9a. The conductivity results (Figure 9b) confirm that all emulsions were w/o. Emulsions are completely stable to coalescence except the one at the highest salt concentration where separated water can be seen. In contrast, their stability to sedimentation increases with salt concentration as illustrated in Figure S6. Figure S7 depicts the decrease in the average droplet diameter with an increasing salt concentration until reaching a plateau value of around 30 μm. By using larger particles (*d* = 3.5 μm) with comparable surface charge density (−129 mC/m^2^) to the smaller ones, particles were clearly observed adsorbing at droplet interfaces by microscopy (Figure S8). The contact angles of 3.5 μm particles at the planar water–dodecane interface are shown in Figure 10 as a function of TPeAB concentration. The value without salt is 117 ± 2°, and an increase in salt concentration results in a progressive reduction of the contact angle to 108 ± 2°. It thus remains above 90° even at the highest salt concentration, consistent with preferred emulsions being w/o. However, we predict that o/w emulsions can be stabilized if the contact angle of particles can be reduced to <90° somehow or if more hydrophilic latex particles are used as a stabilizer. It has been reported that carboxylate-modified PS latex particles with 5.5 × 10^6^ COOH groups per particle (θ > 90°) give w/o emulsions, whereas similar particles of higher charge density equal to 3.5 × 10^7^ COOH groups per particle (θ < 90°) give o/w emulsions regardless of salt concentration.^25^

**Figure 11:**
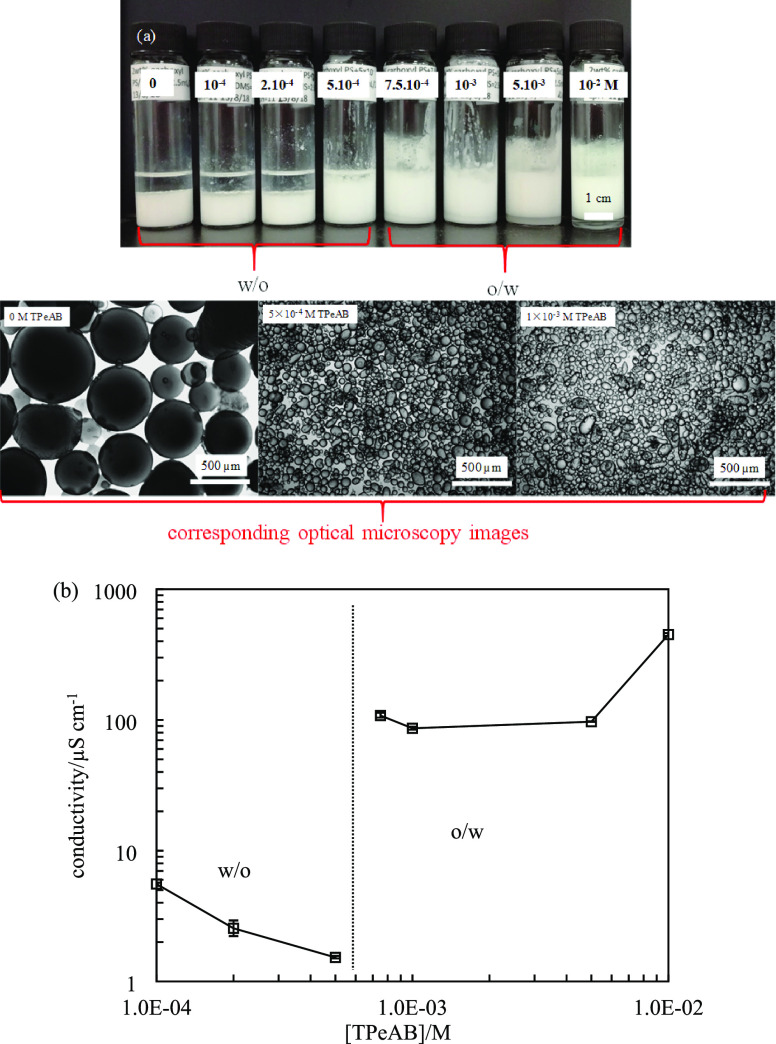
Appearance and selected optical microscopy images of 1 cS PDMS-water emulsions stabilized by carboxyl latex particles (diameter = 0.2 μm) of various concentrations (given in M) of TPeAB at pH = 11. (b) Conductivity and type of emulsions in (a).

The results above indicate that by adding an organic electrolyte to water, we are able to decrease the hydrophobicity of PS particles. However, the magnitude of the change in contact angle is not significant to tune the particle wettability from predominantly hydrophobic (θ > 90°) to predominantly hydrophilic (θ < 90°), promoting o/w emulsion formation. To further decrease the three-phase contact angle of carboxyl latex particles, 1 cS PDMS (containing small oligomers) with slightly higher polarity than dodecane was used as oil. As shown in Figure 11a, emulsions are w/o at [TPeAB] ≤ 5 × 10^–4^ M exhibiting low conductivity (<6 μS cm^–1^). Transitional phase inversion to o/w emulsions then occurs at a higher salt concentration where much higher conductivities (>80 μS cm^–1^) are measured (Figure 11b). Although all emulsions are stable to coalescence, w/o emulsions at low salt concentrations are partially unstable to sedimentation and o/w emulsions at high salt concentrations are partially unstable to creaming (Figure S9). At the highest salt concentration (1 × 10^–2^ M), the resolved water is clear, indicating that the particles initially in the aqueous phase transfer to droplet interfaces after homogenization; indeed, non-spherical droplets are visible, arising from the jamming of particles. As can be seen in Figure S10, the droplets of w/o emulsions are large, whereas much smaller droplets are obtained in o/w emulsions.

**Figure 12:**
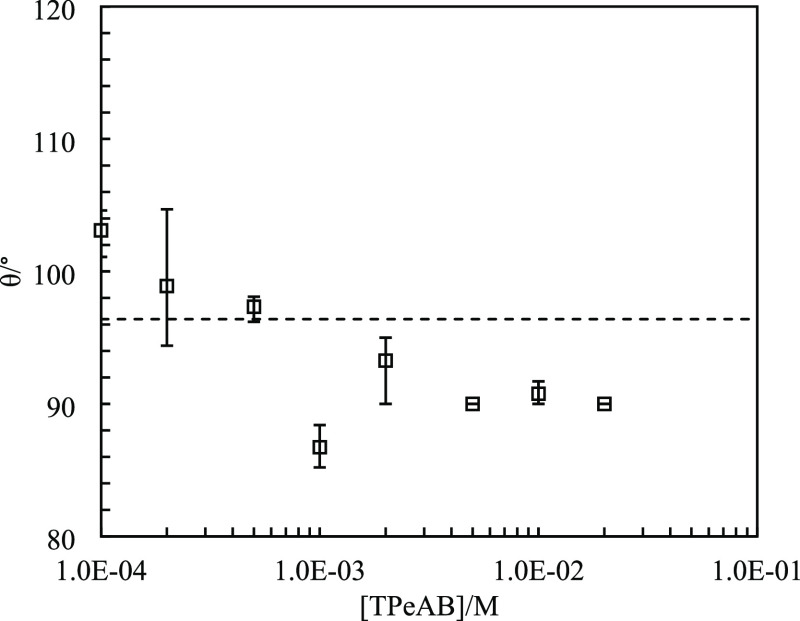
Three-phase contact angle (θ) of carboxyl latex particles (diameter = 3.5 μm) at the 1 cS PDMS–water interface *vs* [TPeAB] at pH = 11. The dashed line indicates angle without salt.

The contact angles of carboxyl latex particles at the planar 1 cS PDMS–water interface are given in Figure 12. As can be seen, the values at TPeAB ≤ 5 × 10^–4^ M are far above 90°, consistent with formation of w/o emulsions. In contrast, at 1 × 10^–3^ M TPeAB where phase inversion occurs, the contact angle is approximately 87° and it remains around 90° for higher salt concentrations. Emulsions of w/o at low salt concentrations are of the Bancroft type as particles are hydrophobic. However, at higher salt concentrations, whether o/w emulsions should be regarded as the anti-Bancroft type is worth discussing as the particle contact angle is now ∼90°. The formation of o/w emulsions may be because particles were initially dispersed in water. Binks and Lumsdon reported that silica particles of intermediate hydrophobicity preferentially stabilize o/w emulsions if they are initially dispersed in water and w/o emulsions if they contact oil first, and they argued in terms of the hysteresis in contact angle either side of 90° depending on which liquid meets particles first.^43^

### Systems with Amidine Latex Particles

**Figure 13:**
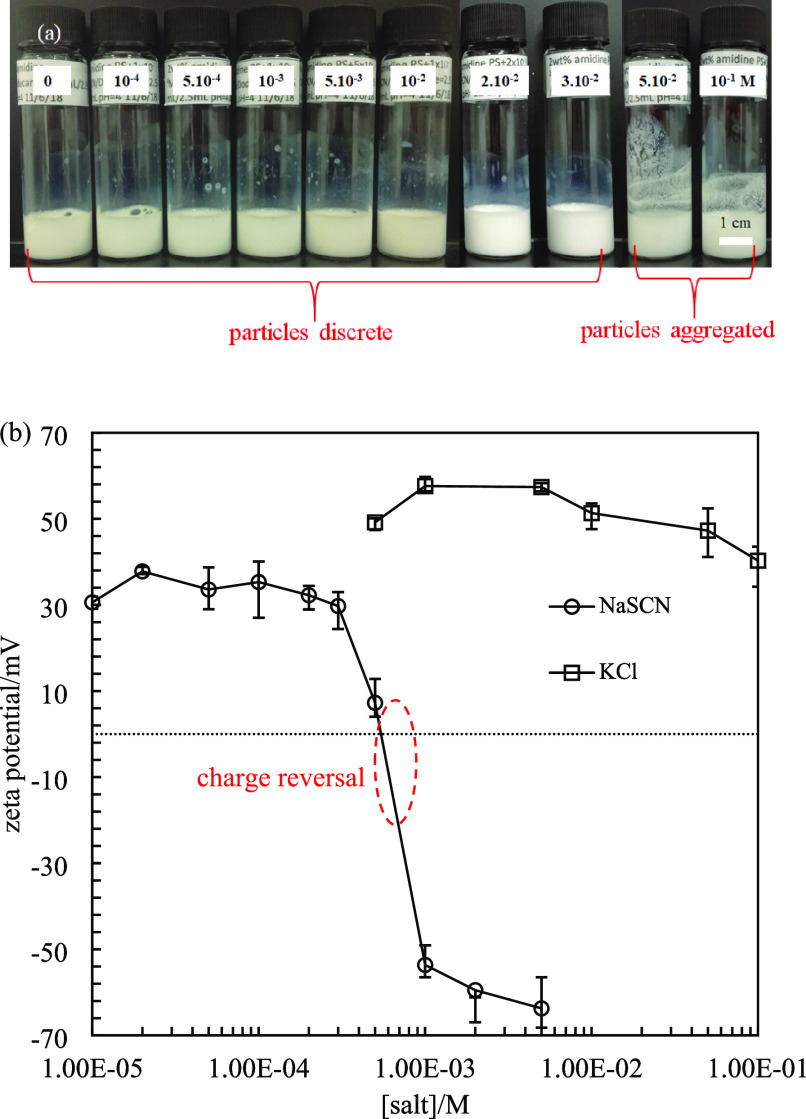
(a) Appearance of 2 wt % amidine latex particle dispersions (diameter = 0.2 μm) at various concentrations of NaSCN at pH = 4 taken immediately after preparation. (b) Zeta potential of 0.0004 wt % amidine latex particles in NaSCN or KCl at pH = 4.

Amidine (C(*NH*)NH_2_) PS latex particle dispersions in sodium thiocyanate (NaSCN) solutions were prepared at pH = 4, where particles are positively charged due to protonation of the amidine groups. Figure 13a shows the appearance of these dispersions, in which particles are discrete up to 5 × 10^–2^ M NaSCN. Above this concentration, large particle aggregates were observed by optical microscopy (Figure S11). The zeta potential of 0.0004 wt % amidine PS latex particles in water is +45 mV (Figure 13b). A slight maximum in zeta potential was observed in KCl solutions, consistent with the literature.^44^ Addition of 1 × 10^–5^ M NaSCN decreases the zeta potential to +30 mV, after which it remains constant until a sharp decrease occurs at 3 × 10^–4^ M NaSCN. Charge reversal from positive to negative was observed between 5 × 10^–4^ M and 1 × 10^–3^ M NaSCN. A similar charge reversal of PS latex particles possessing amine groups on their surface induced by SCN^–^ ions has been reported before, and the ion-specific effect accounted for this phenomenon.^8,19^ Kosmotropic ions such as Cl^–^ can hardly induce charge reversal because they are highly hydrated and do not adsorb on particle surfaces, whereas chaotropic SCN^–^ ions are poorly hydrated and can interact strongly with the hydrophobic PS particle surface, even inducing charge reversal.^14^ It is worth noting that amidine particles do not aggregate significantly until addition of 5 × 10^–2^ M NaSCN or more, far above the IEP of the particles.^45^ This is also due to the strong adsorption of SCN^–^ ions. A short range repulsive force originating from particles surrounded by SCN^–^ ions hinders their aggregation. Reversible aggregation of amidine latex particles in NaSCN solutions has been reported, where clusters with an average of only 2.45 particles were detected in 0.6 M salt.^46^

**Figure 14:**
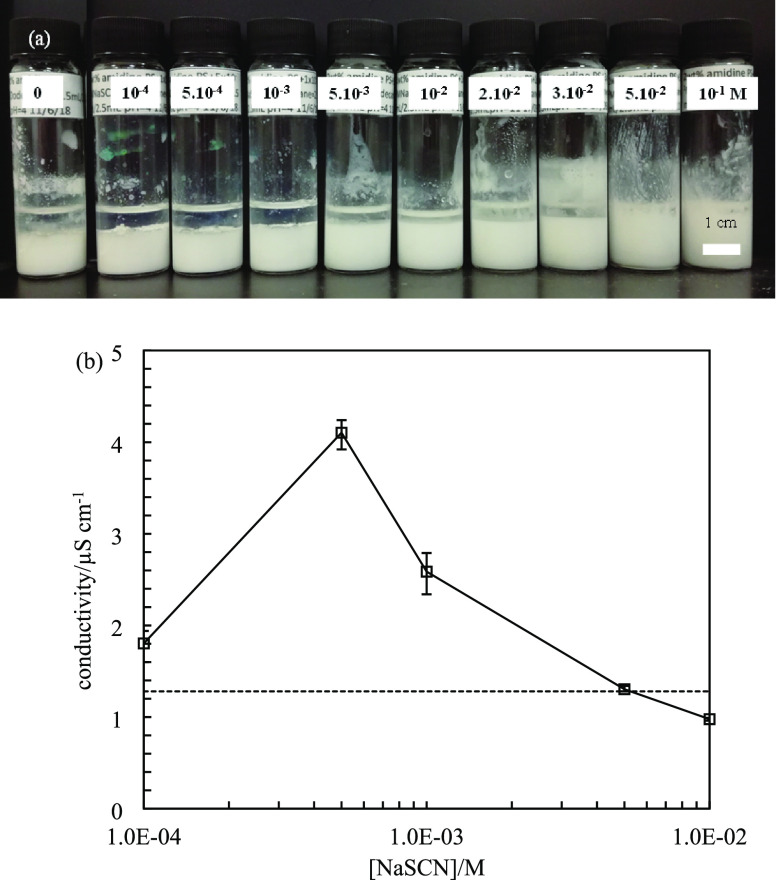
(a) Appearance of water-in-dodecane emulsions (*ϕ_w_* = 0.5) stabilized by 2 wt.% amidine latex particles (diameter = 0.2 μm) at various concentrations of NaSCN at pH = 4 taken one week after preparation. (b) Conductivity of emulsions in (a) measured immediately after homogenization. The dashed line represents the conductivity of emulsion without salt.

The appearance of emulsions stabilized by 2 wt % amidine latex particles is shown in Figure 14a, and all were w/o verified by conductivity measurements (Figure 14b). Amidine latex particles here are not hydrophilic enough to stabilize o/w emulsions, which may be due to the low density of surface groups. The w/o emulsions are stable to coalescence, and *f*_o_, representative of sedimentation, decreases with salt concentration (Figure S12). Figure S13 includes optical microscopy images of certain emulsions, while Figure S14 is a plot of the average droplet diameter as a function of [NaSCN]. It is thus seen that charge reversal of the particles in water has no dramatic influence on these emulsions and does not lead to emulsion phase inversion either.

**Figure 15:**
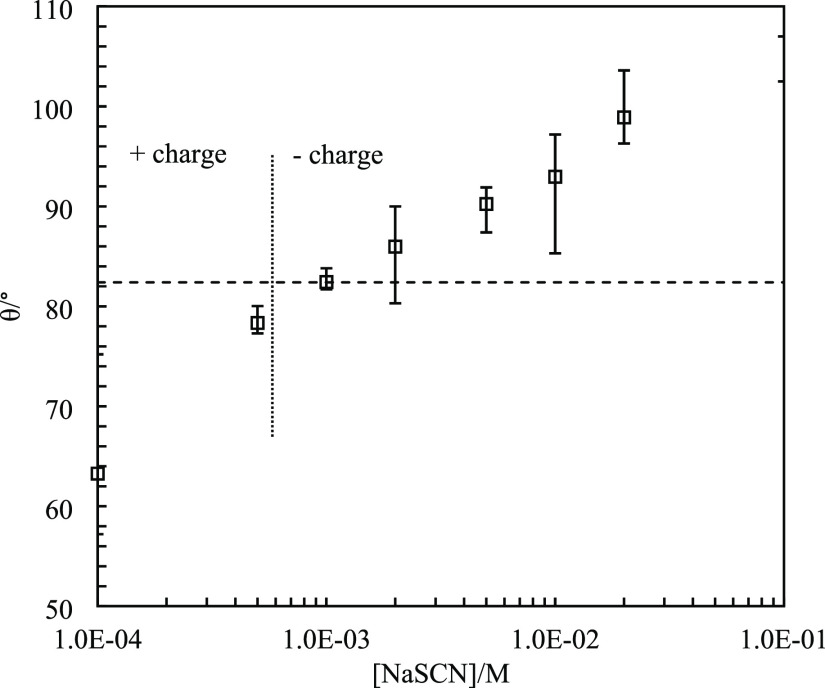
Three-phase contact angle (θ) of monodisperse amidine latex particles (diameter = 1 μm) at the dodecane–water interface in the presence of NaSCN at pH = 4. The horizontal dashed line indicates the contact angle without salt.

The contact angles of 1 μm amidine latex particles at the dodecane–water interface are shown in Figure 15. A value of 82° was obtained for the system without salt, while a slight decrease was obtained upon addition of 1 × 10^–4^ M NaSCN. A subsequent increase in salt concentration resulted in a gradual increase in contact angle to 105°. The initial decrease is reasonable as due to ion specificity, SCN^–^ ions accumulate on amidine latex particle surfaces, breaking the water structure around them.^17^ The subsequent increase is because poorly hydrated ions replace water molecules around particles. A schematic of the possible arrangement of SCN^–^ ions on the surface of these particles is shown in Figure S4b. The contact angle results indicate that the particles should preferentially stabilize o/w emulsions at low salt concentrations (<5 × 10^–3^ M) where contact angles are below 90° and w/o emulsions at higher salt concentrations. However, w/o emulsions are obtained at all salt concentrations. The reason may lie in the method used to measure the contact angle. Gellan gum is a polysaccharide with a carboxylate group from glucuronic acid in its repeat unit**.** At high temperatures, it dissolves in water, forming single coils in which disordered chains are highly extended due to electrostatic repulsion between ionized carboxylate groups.^33^ At this stage (where particles are spread at the oil–water interface), gellan molecules can potentially adsorb on positively charged amidine latex particles. This adsorption may result in a reduced hydrophobicity of the particles as gellan molecules are very hydrophilic. In this case, the GTT may not be suitable for measurements involving positively charged particles unless another non-adsorbing gel is used.

## Conclusions

Pickering emulsions stabilized by PS latex particles in the presence of specific electrolytes were investigated. The particles used are partially hydrophobic but are initially dispersed in water due to their charged surface groups (sulfate, carboxyl, and amidine). The presence of TPeAB in aqueous dispersions of sulfate and carboxyl latex particles reduces their surface charge and leads to charge reversal in the former. However, the type of emulsion with non-polar oils such as dodecane stabilized by these particles is dominated by the hydrophobic PS fraction on particle surfaces. Consequently, preferred emulsions are w/o and the contact angles of particles at the planar oil–water interface are >90°. The surface charge plays a minor role in determining emulsion type. The adsorption of TPeA^+^ ions on particle surfaces slightly decreases their hydrophobicity, resulting in an increase in emulsion stability to both sedimentation and coalescence. Charge reversal of sulfate latex particles induces no specific influence on the type or stability of emulsions.

Transitional phase inversion can be achieved however with carboxyl latex particles and 1 cS PDMS oil with emulsions inverting from w/o to o/w as the concentration of TPeAB increases. The contact angle of particles is >90° where w/o emulsions form and around 90° where o/w emulsions form. For systems containing amidine latex particles, addition of NaSCN leads to charge reversal. Emulsions of dodecane are all w/o, although the contact angle of particles increases through 90° with an increasing salt concentration. Charge reversal of particles has little influence on emulsions. Adsorption of SCN^–^ ions is driven by an ion-specific effect. Poorly hydrated SCN^–^ ions break the structure of water around particles, increasing their hydrophobicity.

## Supporting Information Available

SEM of polystyrene particles, photos of the peeling elastomer layer from gelled gellan gum, size distributions of sulfate particles, schematic of counterions adsorbing onto particles, diameter and microscopy images of carboxyl particles, stability of water-in-dodecane emulsions by carboxyl particles, droplet diameter of water-in-dodecane emulsions stabilized by carboxyl particles, microscopy image of carboxyl particles at the droplet surface, stability of PDMS–water emulsions stabilized by carboxyl particles, droplet diameter of PDMS–water emulsions stabilized by carboxyl particles, diameter and microscopy images of amidine particles, stability of water-in-dodecane emulsions stabilized by amidine particles, and microscopy images and droplet size of water-in-dodecane emulsions stabilized by amidine particles (PDF)
