# Supplementary material for: Pickering Emulsions Stabilized by Polystyrene Particles Possessing Different Surface Groups
Source: Langmuir. 2022 Jan 14;38(3):1079–89. doi: 10.1021/acs.langmuir.1c02648 (PMC9171833; doi:10.1021/acs.langmuir.1c02648)

# ESI

# PICKERING EMULSIONS STABILISED BY POLYSTYRENE PARTICLES POSSESSING DIFFERENT SURFACE GROUPS

Raojun Zheng and Bernard P. Binks*

*Department of Chemistry, University of Hull, Hull. HU6 7RX. U.K.*

No. of pages: 15

No. of Figures: 14

**Figure S1.** SEM images of (a) sulfate latex particles, (b) amidine latex particles and (c) carboxyl latex particles. Particles were coated by gold. Mean diameter calculated from the images is (a) 1.80 ± 0.09 μm, (b) 1.04 ± 0.02 μm and (c) 3.56 ± 0.55 μm.

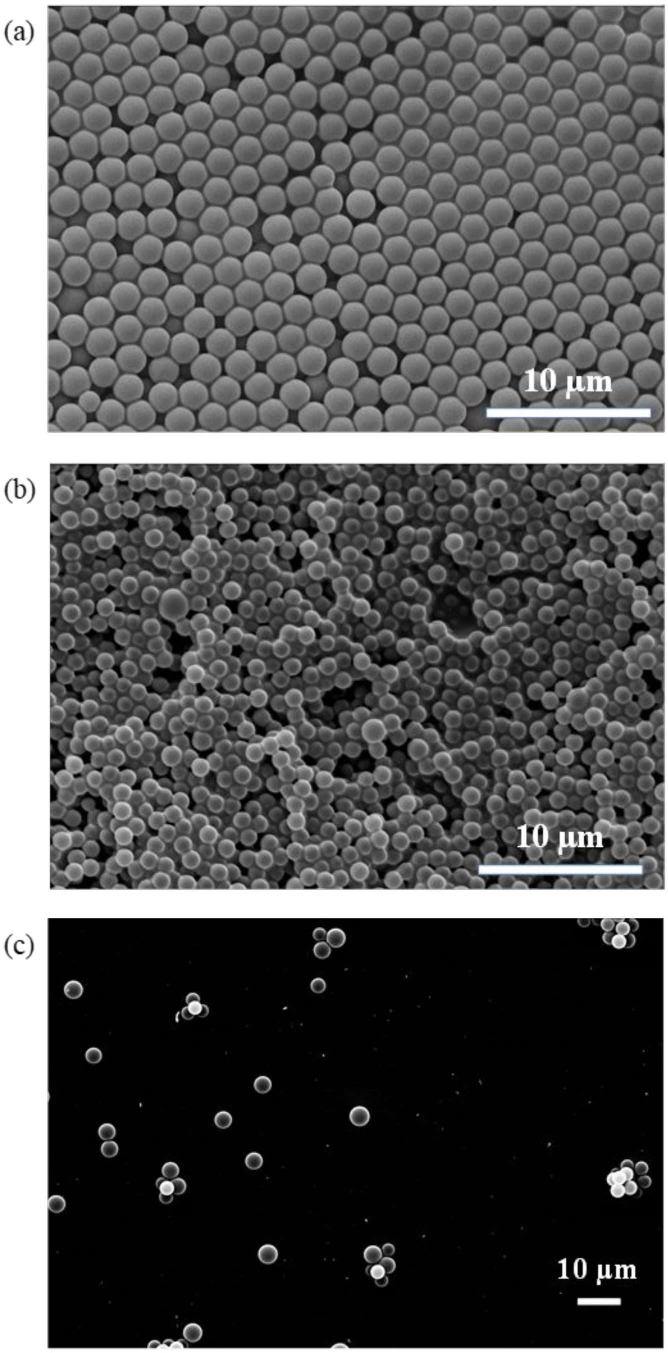

**Figure S2.** (a) Peeling PDMS Sylgard 184 layer with sulfate latex particles (diameter = 2 μm) from gelled aqueous phase at pH = 4. (b) Appearance of PDMS Sylgard 184 layer with sulfate latex particles.

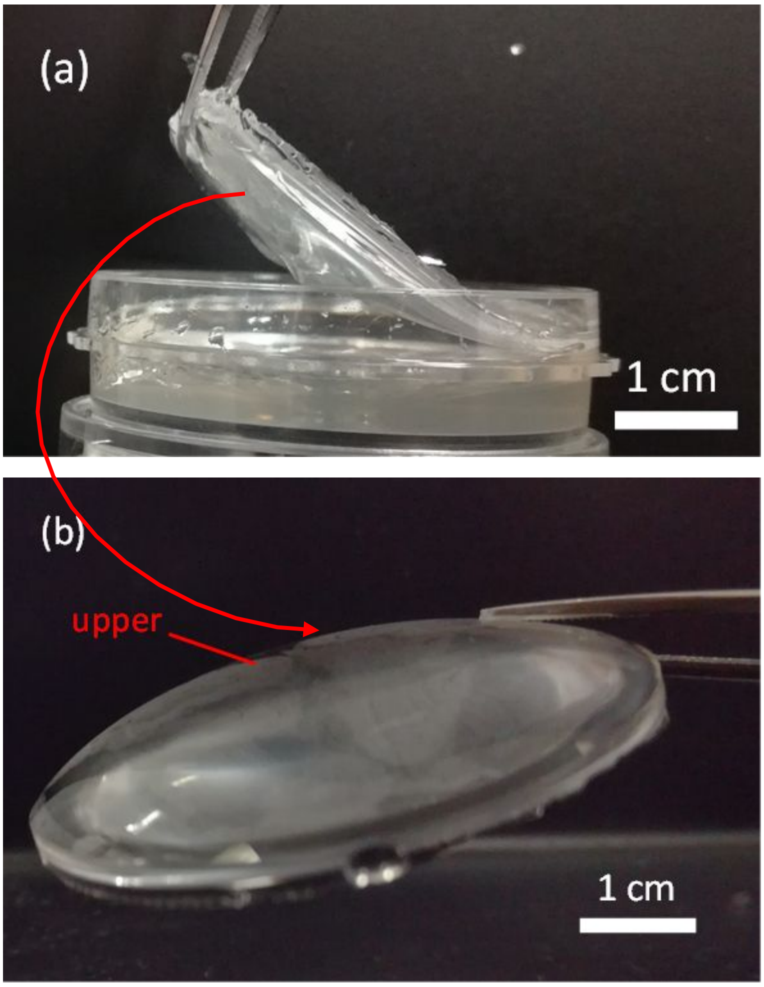

**Figure S3.** Size distribution of sulfate latex particles (quoted diameter = 0.2 μm) in the presence of (a) 0 M, (b) $1{\times}10^{-4}$ M and (c) $1{\times}10^{-3}$ M TPeAB at pH = 4.

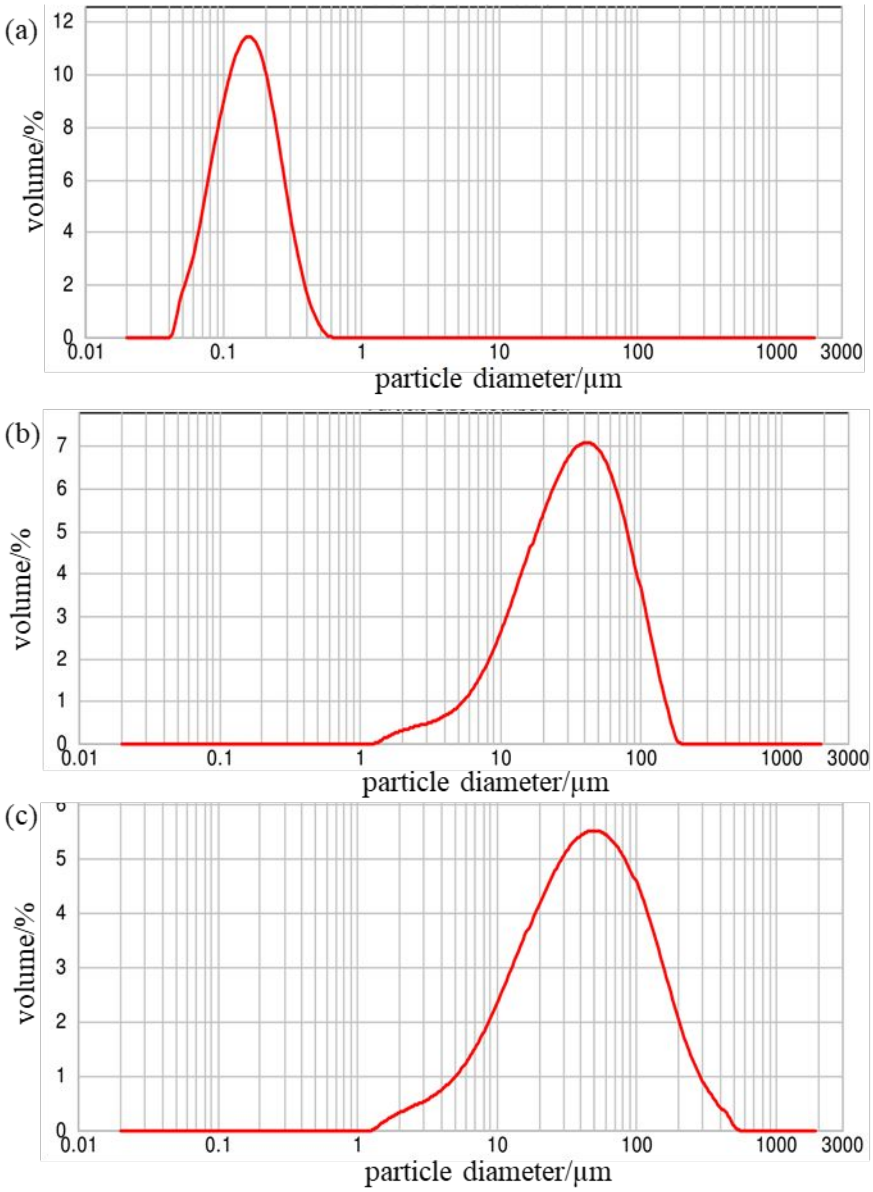

**Figure S4.** Schematic of possible adsorption of (a) $TPeA^+$ ions onto sulfate latex particles and (b) $SCN^-$ ions onto amidine latex particles.

(a)

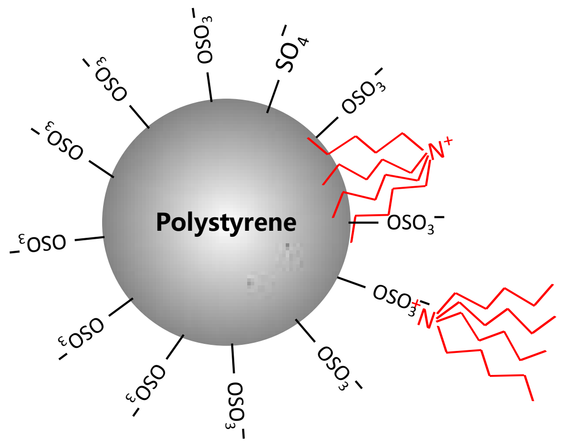


(b)

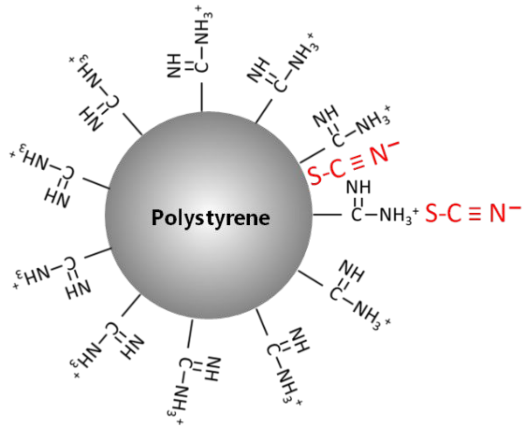

**Figure S5.** (a) Average diameter of carboxyl latex particles at different [TPeAB] at pH = 11. (b) Optical microscopy images of 2 wt.% carboxyl latex particles (diameter = 0.2 μm) in the presence of TPeAB at pH = 11.

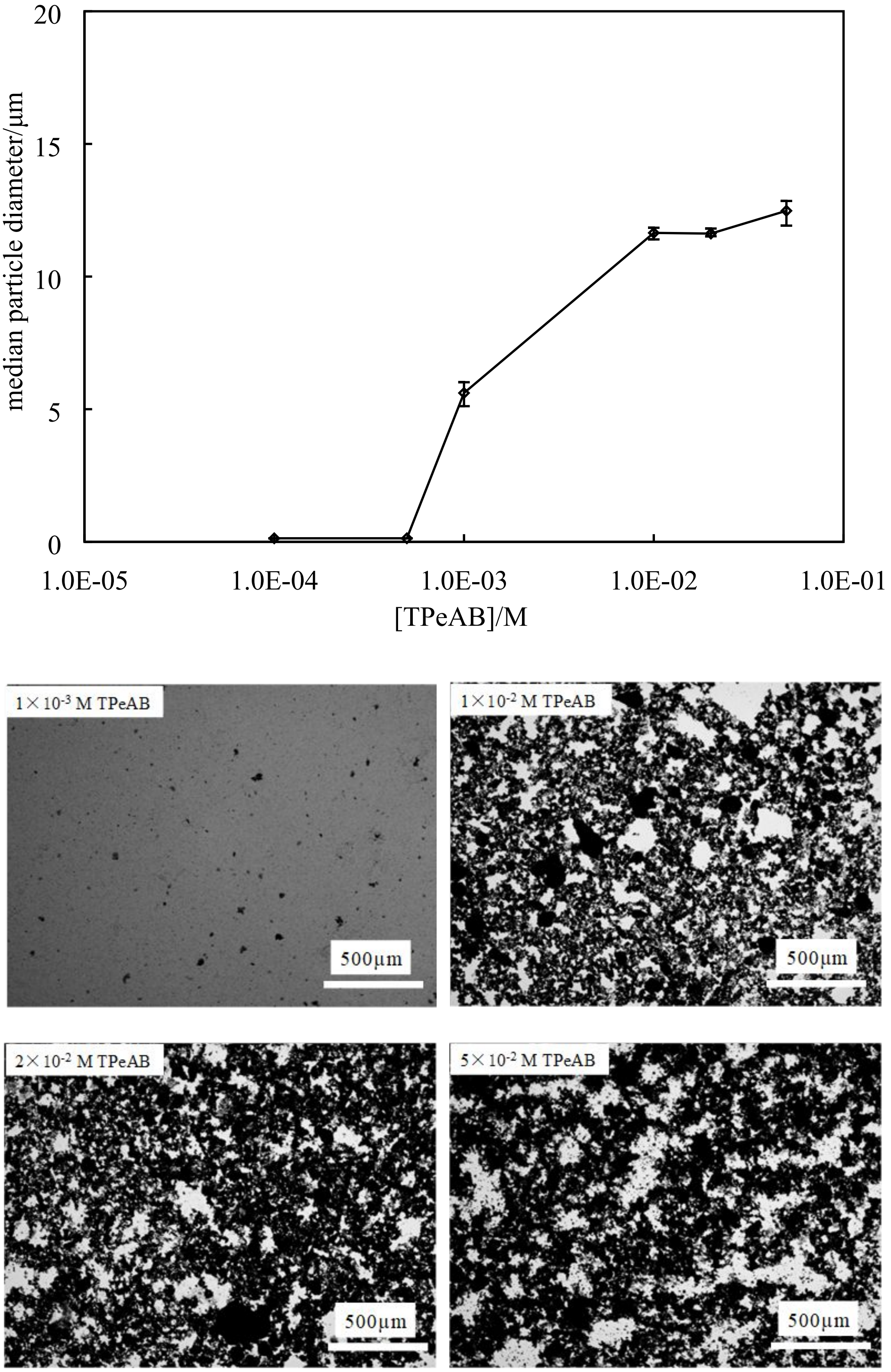

**Figure S6.** Stability to sedimentation ($f_o$) and coalescence ($f_w$) of water-in-dodecane emulsions ($\phi_w$ = 0.5) stabilized by 2 wt.% carboxyl latex particles (diameter = 0.2 μm) at different [TPeAB] at pH = 11, measured one week after preparation. Dashed line indicates $f_o$ of emulsion without salt.

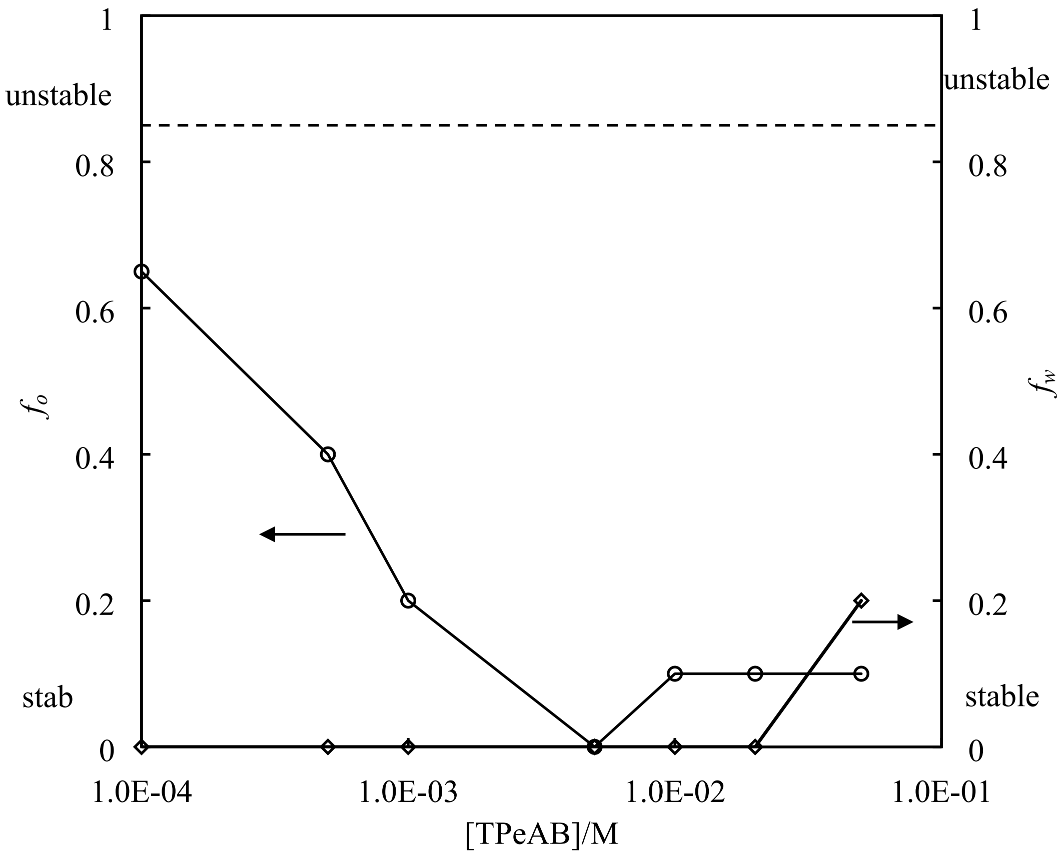

**Figure S7.** Average droplet diameter of water-in-dodecane emulsions ($\phi_w$ = 0.5) stabilized by 2 wt.% carboxyl latex particles (diameter = 0.2 μm) against [TPeAB] at pH = 11. Dashed line represents the droplet diameter of emulsion without salt.

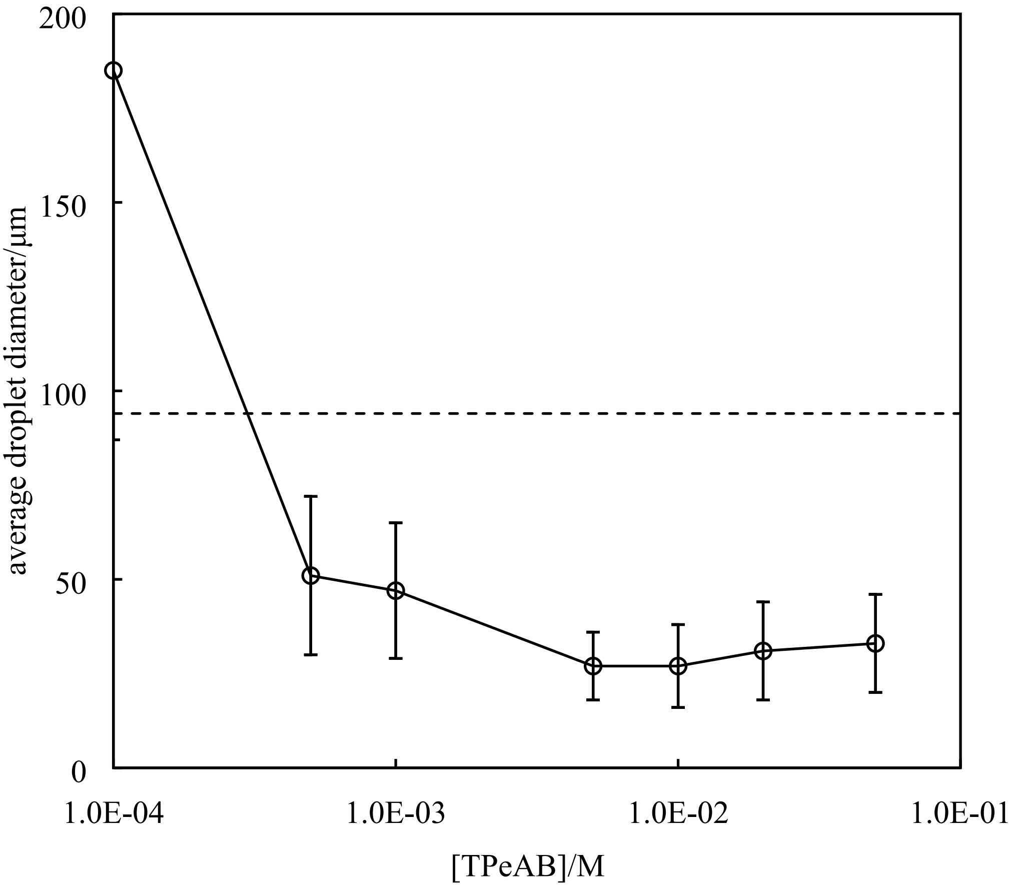

**Figure S8.** Optical microscopy image of water-in-dodecane emulsion stabilised by 2 wt.% carboxyl latex particles (diameter = 3.5 μm) in the presence of $5\times10^{-4}$ M TPeAB at pH = 11.

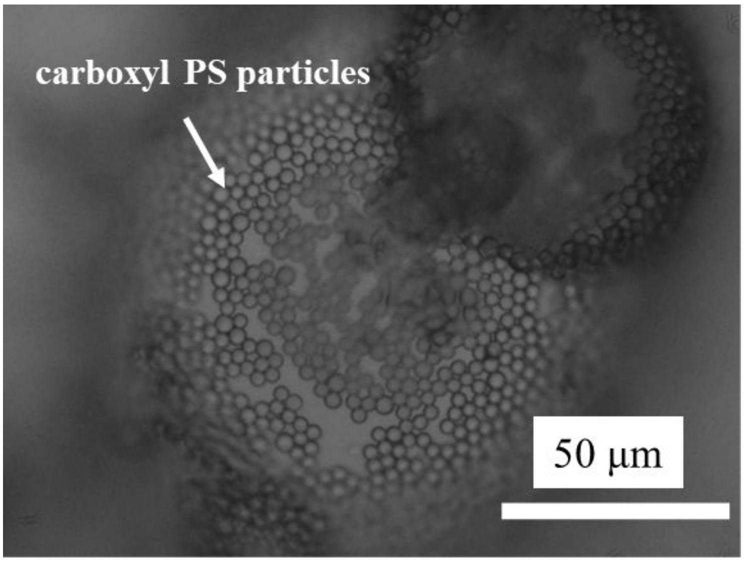

**Figure S9.** $f_o$ (left hand ordinate, circles) and $f_w$ (right hand ordinate, diamonds) of 1 cS PDMS-water emulsions ($\phi_w$ = 0.5) stabilized by 2 wt.% carboxyl latex particles against [TPeAB] at pH = 11, measured 1 week after preparation. Horizontal dashed line indicates the stability of w/o emulsion without salt.

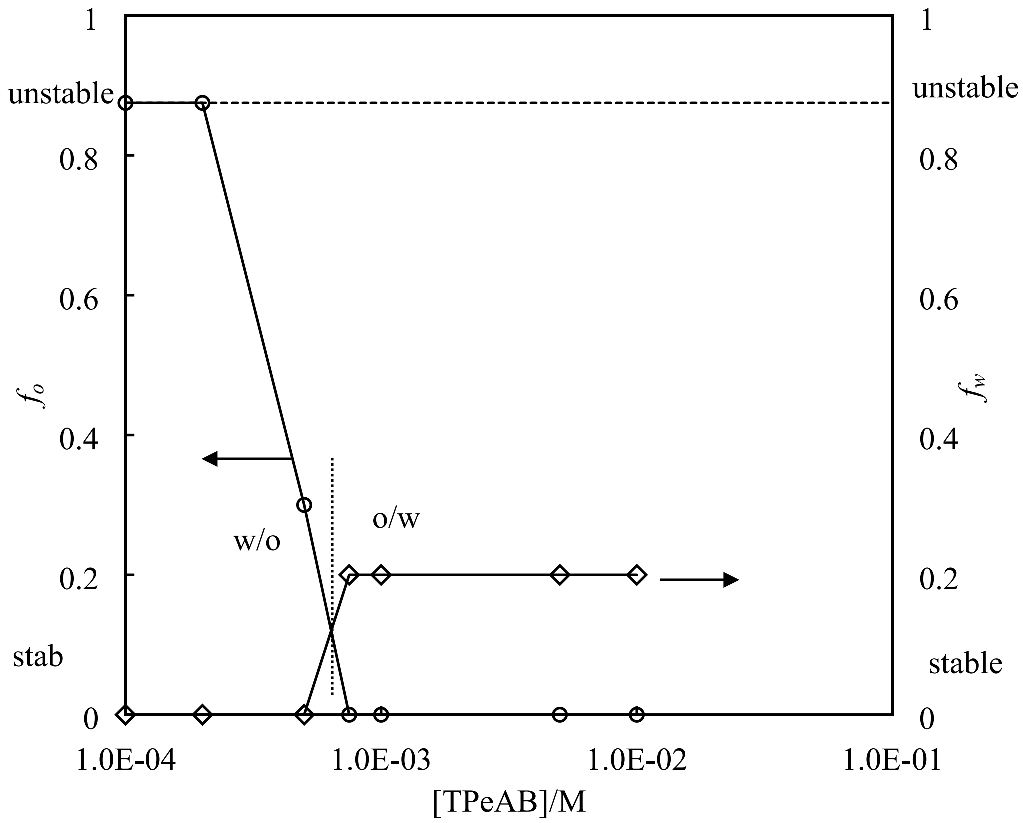

**Figure S10.** Average droplet diameter and type of 1 cS PDMS-water emulsions ($\phi_w$ = 0.5) stabilized by 2 wt.% carboxyl latex particles at different [TPeAB] at pH = 11. Dashed line indicates droplet diameter without salt.

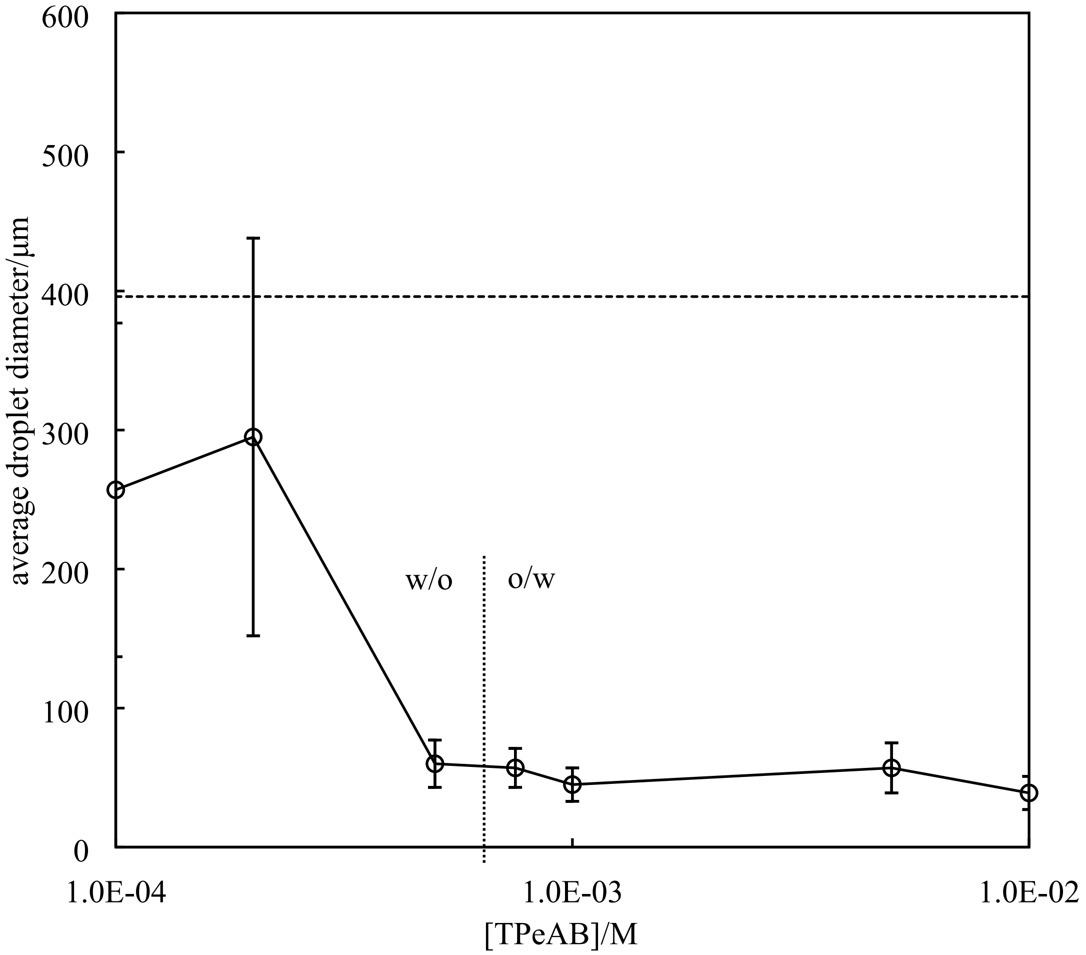

**Figure S11.** (a) Average diameter of amidine latex particles at different [NaSCN] at pH = 4, (b) corresponding optical microscopy images.

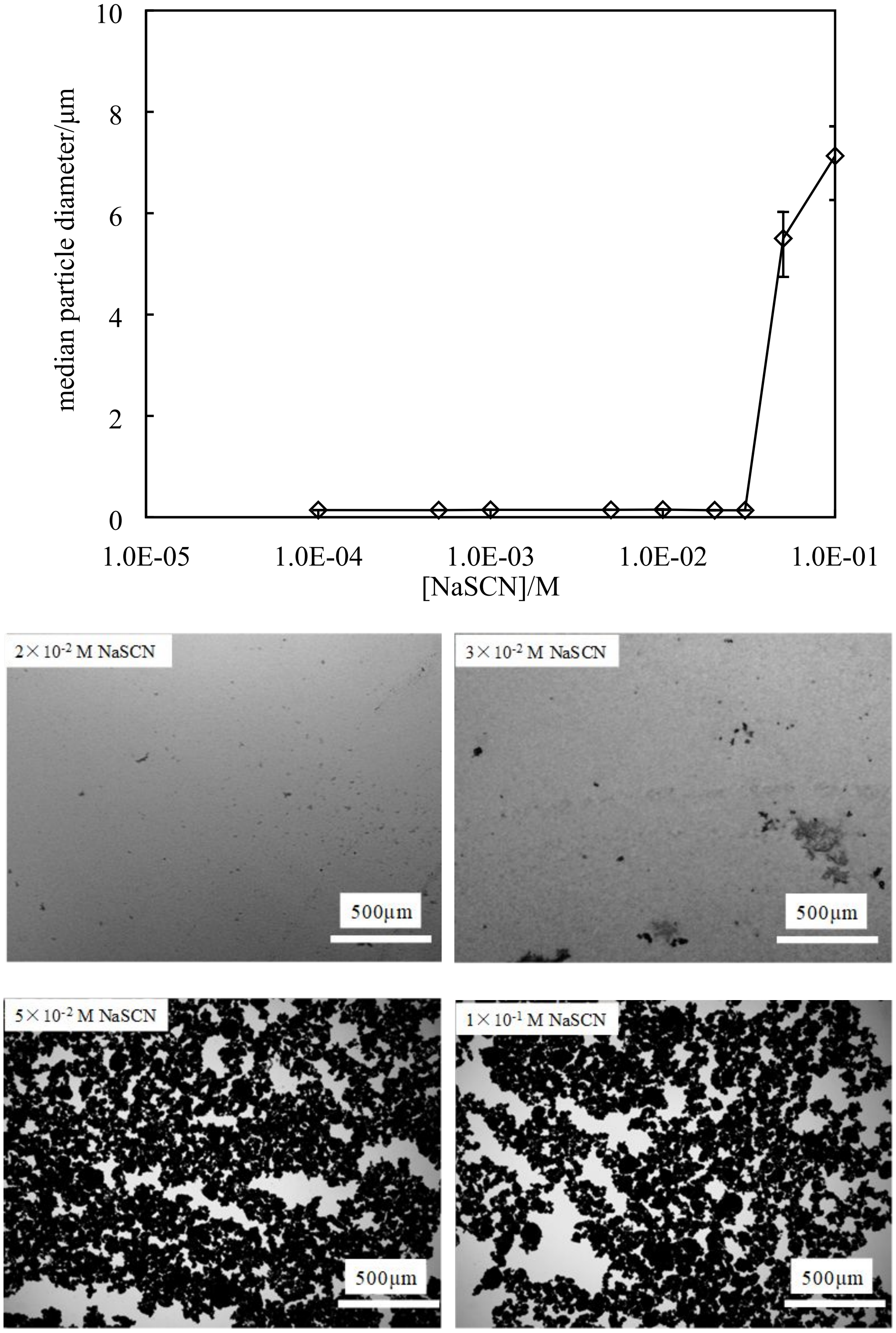

**Figure S12.** Stability to sedimentation ($f_o$) and coalescence ($f_w$) of water-in-dodecane emulsions ($\phi_w$ = 0.5) stabilized by 2 wt.% amidine latex particles (diameter = 0.2 μm) *vs* [NaSCN] at pH = 4, measured one week after preparation. Dashed line indicates $f_o$ of emulsion without salt; $f_w$ of emulsions is zero at all salt concentrations.

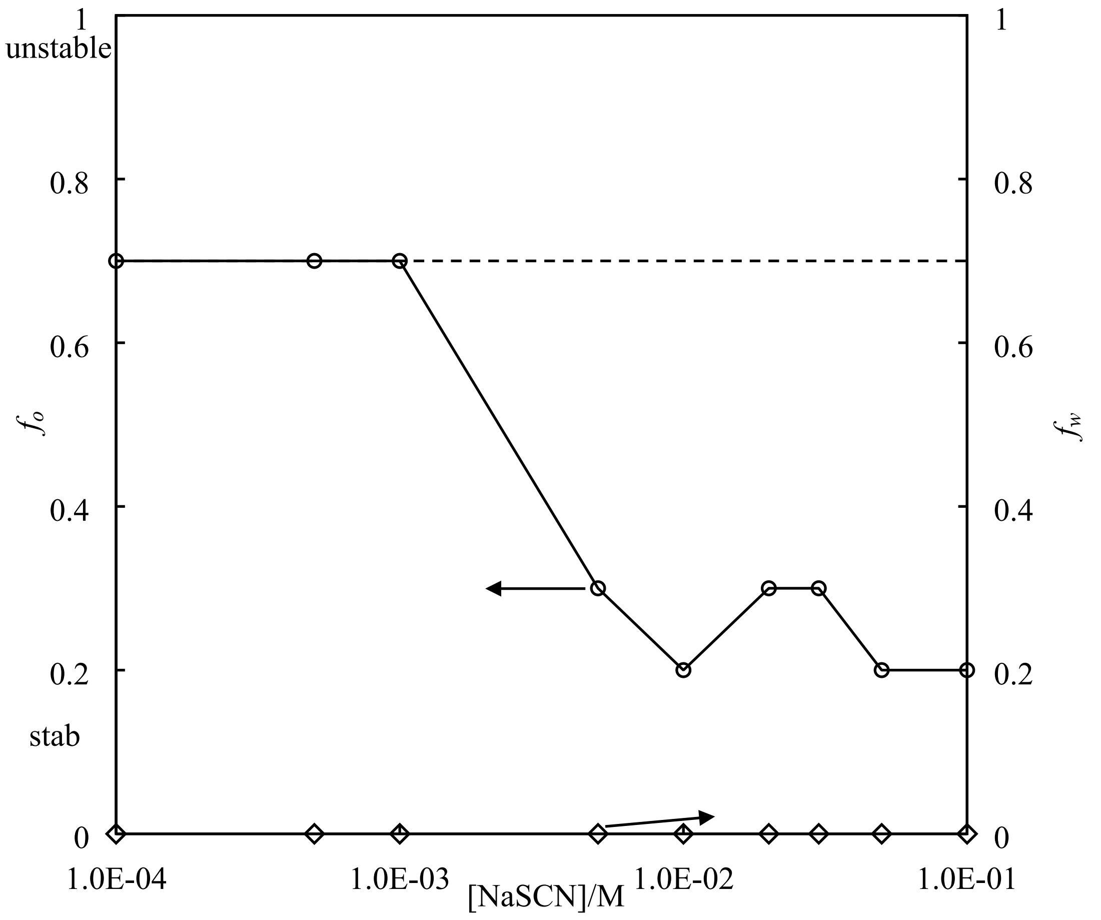

**Figure S13.** Optical micrographs of water-in-dodecane emulsions ($\phi_w = 0.5$) stabilized by 2 wt.% amidine latex particles at different [NaSCN] at pH = 4, taken 24 h after preparation.

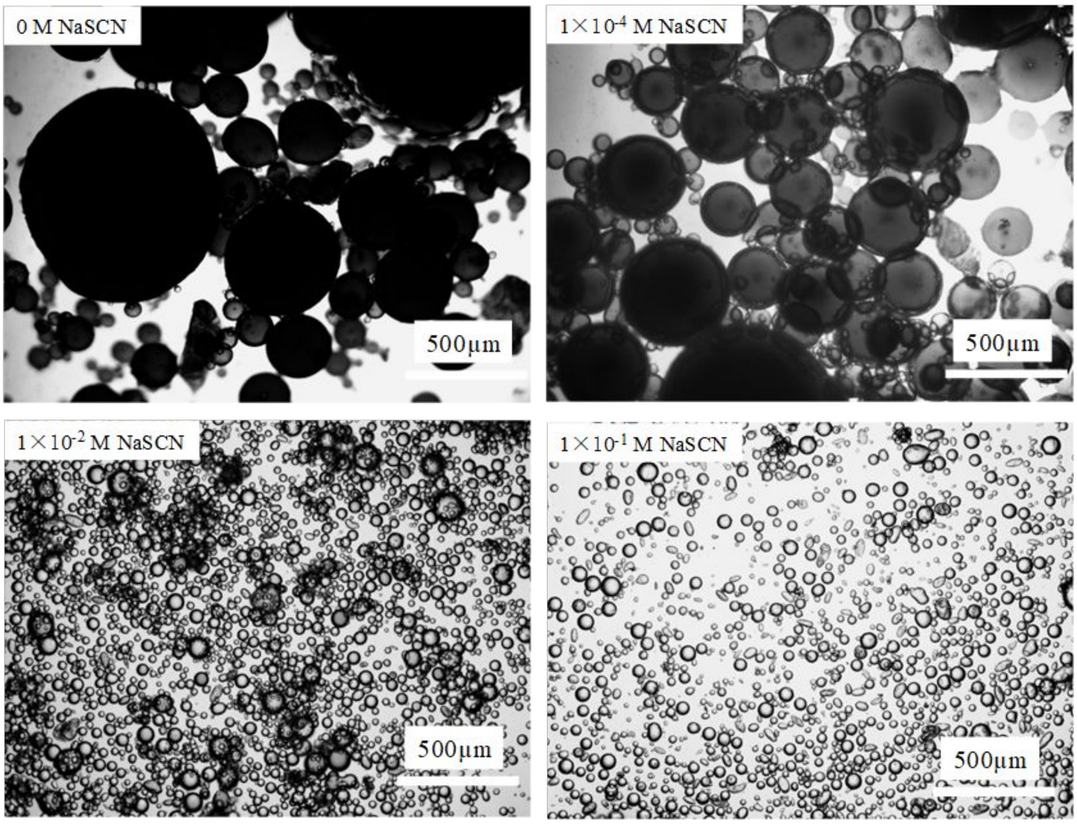

**Figure S14.** Average droplet diameter of water-in-dodecane emulsions ($\phi_w$ = 0.5) stabilized by 2 wt.% amidine latex particles (diameter = 0.2 μm) *vs* [NaSCN] at pH = 4. Dashed line represents average droplet diameter of emulsion without salt.

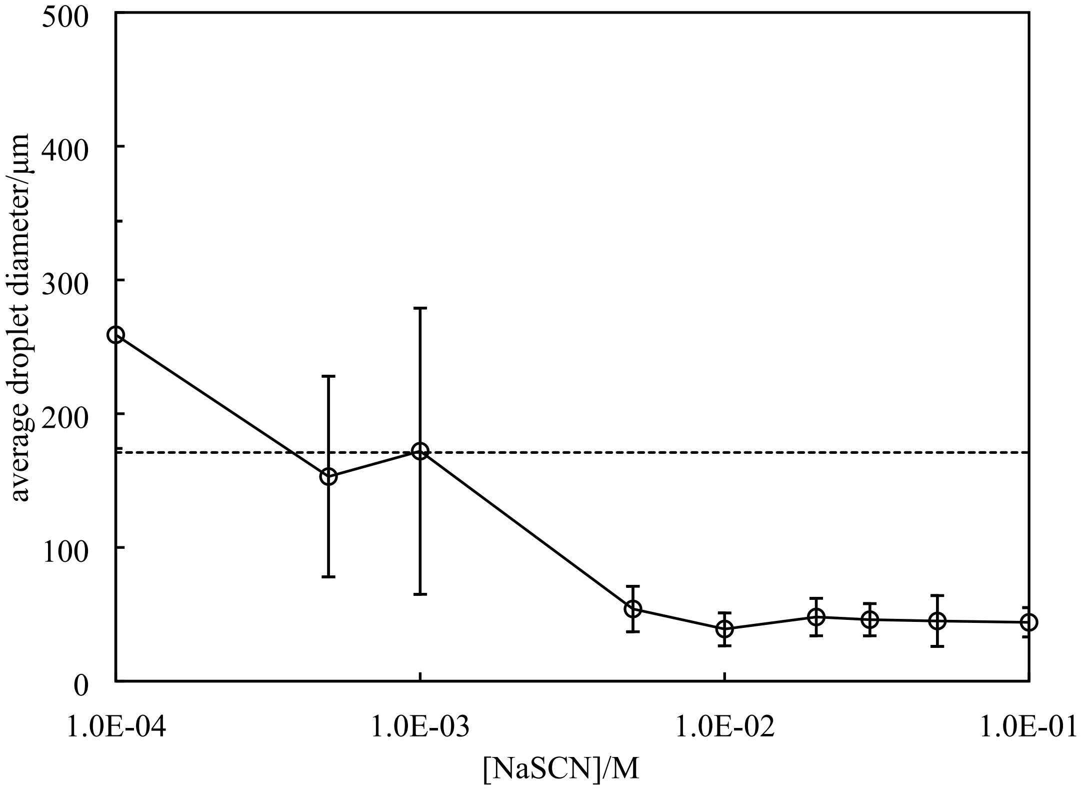

Supplement: Supplementary file 1 — la1c02648_si_001.pdf [file la1c02648_si_001.pdf]
